# Anomalous visual experience is linked to perceptual uncertainty and visual imagery vividness

**DOI:** 10.1007/s00426-020-01364-7

**Published:** 2020-05-31

**Authors:** Johannes H. Salge, Stefan Pollmann, Reshanne R. Reeder

**Affiliations:** 1 Department of Experimental Psychology, Institute of Psychology, Otto-Von-Guericke University, Magdeburg, Germany; 2 Center for Behavioral Brain Sciences, Magdeburg, Germany; 3 Beijing Key Laboratory of Learning and Cognition and School of Psychology, Capital Normal University, Beijing, China

## Abstract

An imbalance between top-down and bottom-up processing on perception (specifically, over-reliance on top-down processing) can lead to anomalous perception, such as illusions. One factor that may be involved in anomalous perception is visual mental imagery, which is the experience of “seeing” with the mind’s eye. There are vast individual differences in self-reported imagery vividness, and more vivid imagery is linked to a more sensory-like experience. We, therefore, hypothesized that susceptibility to anomalous perception is linked to individual imagery vividness. To investigate this, we adopted a paradigm that is known to elicit the perception of faces in pure visual noise (pareidolia). In four experiments, we explored how imagery vividness contributes to this experience under different response instructions and environments. We found strong evidence that people with more vivid imagery were more likely to see faces in the noise, although removing suggestive instructions weakened this relationship. Analyses from the first two experiments led us to explore confidence as another factor in pareidolia proneness. We, therefore, modulated environment noise and added a confidence rating in a novel design. We found strong evidence that pareidolia proneness is correlated with uncertainty about real percepts. Decreasing perceptual ambiguity abolished the relationship between pareidolia proneness and both imagery vividness and confidence. The results cannot be explained by incidental face-like patterns in the noise, individual variations in response bias, perceptual sensitivity, subjective perceptual thresholds, viewing distance, testing environments, motivation, gender, or prosopagnosia. This indicates a critical role of mental imagery vividness and perceptual uncertainty in anomalous perceptual experience.

**Electronic supplementary material:**

The online version of this article (10.1007/s00426-020-01364-7) contains supplementary material, which is available to authorized users.

## Introduction

Perception is the mental interpretation of external sensory stimuli—an individualized experience that requires a delicate balance between top-down (cognitive) and bottom-up (environmental) sources of information. A tip in that balance toward internal sources can result in differences in perceptual biases and even errors. One internal source of sensory information is visual mental imagery, which is the ability to “see” with the mind's eye. There is wide individual variability in visual imagery vividness (Kosslyn, Brunn, Cave, & Wallach, 1984). Several studies have shown that if an individual's imagery is strong (i.e., more sensory-like), it will activate sensory brain regions to a larger extent than if an individual's imagery is weak (Bergmann, Genç, Kohler, Singer, & Pearson, 2016; Cui, Jeter, Yang, Montague, & Eagleman, 2007; Dijkstra, Bosch, & Gerven, 2017; Lee, Kravitz, & Baker, 2012; Olivetti Belardinelli et al., 2009). A higher reliance on sensory brain regions for imagery means a higher neural overlap for imagery and perception. An over-reliance on imagery for perception may contribute to anomalous perceptual experience, such as hallucinations in pathological conditions (Shine et al., 2015).

Hallucinations are proposed to occur when people fail to distinguish between mental imagery and sensory input (also referred to as “reality monitoring” errors: Aleman et al., 2003; Bentall, 1990; Smailes et al., 2020). In line with this, previous studies have found evidence for a link between mental imagery vividness and hallucination proneness in various disorders (Aleman et al., 2000; Aynsworth et al., 2017; Shine et al., 2015). Because most studies of anomalous perception rely on clinical samples, it is impossible to disentangle whether these experiences emerge from a predisposition (i.e., more vivid imagery prior to disorder), or develop comorbidly with pathology (i.e., more vivid imagery following the onset of a disorder). In the current study, we, therefore, addressed this problem by investigating the link between imagery vividness and anomalous perception in a normative sample, prior to (or in the absence of) pathology. We hypothesized that the extent to which an individual relies on mental imagery to inform perceptual decisions (and the likelihood to commit a reality monitoring error) may be linked to individual imagery vividness. To test this hypothesis, we turned to pareidolia.

Pareidolia—the misinterpretation of veridical sensory information—is a type of reality monitoring error that is commonly experienced in the normative population (Smailes et al., 2020; Uchiyama et al., 2012). Pareidolia proneness is also linked to hallucination proneness in pathology (Mamiya et al., 2016; Uchiyama et al., 2012; Yokoi et al., 2014). Visual pareidolia experience is thought to reflect a strong reliance on internally generated visual information for visual processing (Smailes et al., 2020), and can be induced reliably with perceptually ambiguous visual stimulation, such as briefly presented patches of Gaussian noise. In an often-used paradigm by Zhang et al. (2008), subjects are required to detect faces in this noise. Over training trials, real faces become more difficult to detect, until the final training trials contain no faces. In the main experiment, only pure noise images are presented, but subjects reliably report the presence of faces throughout.

Because pareidolia is a subjective experience influenced by top-down factors, we hypothesized that there should be individual variability in the frequency of these percepts. In line with this, we found that pareidolia experiences are reported with varying prevalence across studies that have used the same or similar experimental design (Liu et al., 2014; Rieth, Lee, Lui, Tian, & Huber, 2011; Zhang et al., 2008; Zimmermann, Stratil, Thome, Sommer, & Jansen, 2019). In Zhang et al. (2008), the percentage of face-present responses in 16 subjects ranged from nearly 8% to more than 50% (standard deviation (SD) = 14%). Liu et al. (2014) reported the percentage of face-present responses at over 34%, with a SD of over 15% for 20 subjects. Rieth et al. (2011) reported 39% face-present responses, after excluded nearly 25% of their 229 subjects who reported to see fewer than 5 faces in the whole experiment. At the cortical level, one recent study found that face-selective cortical areas were only activated in 4/9 subjects in this design (Zimmermann et al., 2019). These results clearly show a range of individual differences in pareidolia proneness that has not yet been explained. In the current study, we investigated whether individual imagery vividness (that is, the strength of top-down sensory representations) contributes to this variability.

One problem is that the variability in subject responses reported in previous studies could be attributed to confounding factors in the experimental design. It is possible that some subjects report to see faces in noise simply because they feel pressured to respond positively (e.g. on about 50% of trials), and so lower their response criterion; furthermore, responses may be influenced by bottom-up patterns contained in the noise that happen to appear face-like (Gosselin & Schyns, 2003; Rieth et al., 2011), and some subjects may have a lower “signal detection” criterion than others (Dolgov & McBeath, 2005). Therefore, the aim of the current study was to investigate the role of imagery vividness in pareidolia proneness when controlling for these factors.

We conducted a behavioral replication of Zhang et al. (2008; Exp. 1a), a follow-up to minimize acquiescence response bias (Exp. 1b), and interim analyses to rule out additional response biases and specific bottom-up influences on responses. Following those analyses, we suspected that perceptual uncertainty may also contribute to pareidolia experiences, so we conducted two further experiments to increase (Exp. 2a) and decrease (Exp. 2b) the noisiness of the environment and explore the effects of both imagery vividness and perceptual confidence on pareidolia proneness. Our results support the hypothesis that both vivid imagery and a decrease in perceptual confidence contribute to pareidolia proneness. Importantly, we discuss how these factors contribute to anomalous perceptual experience in the healthy population, and how we might be able to better understand pathological hallucinatory experience from these experiments.^1^

## Experiment 1a: replication

Experiment 1a was a direct replication of Zhang et al. (2008)*.* Here, we were interested to investigate the role of imagery vividness in pareidolia proneness using the same paradigm that has been used in previous studies.

### Methods

#### Subjects

The experiment was performed in the Magdeburg Experimental Laboratory of Economic Research (MaXLab) at Otto-von-Guericke University in Magdeburg, Germany. 50 subjects were recruited via the MaXLab online recruitment tool. All data were collected over 3 testing days, and were analyzed following complete data collection. We obtained a diverse sample of bachelor's and master's students studying engineering, business/economics, social science, psychology, mathematics, medicine, sport science, history, philosophy, and computer science. All subjects provided written, informed consent to take part in the experiment. Subjects were reimbursed for 10 euros for the 1-h experiment, as per laboratory guidelines. The experiment was approved by the Otto-von-Guericke University ethics committee and adhered to the tenets of the Declaration of Helsinki.

8 subjects were excluded due to incomplete data (no responses on a questionnaire and/or the main experiment) or failing to pass the attention check. We had a final group size of 42. Our sample included 20 women, 4 left handed, 5 English speakers, and 37 German speakers, with a mean age of 24.21 years (range 20–35).

#### Apparatus

The experiments were performed on 19-inch Belinea 10 19 20 and Hanns-G HA191 computer monitors with 1280 × 1024 pixel resolution and 60 Hz refresh frequency. There were 46 cm between the monitor and the table edge. Subjects performed the experiment from a free-viewing distance with no chin/head stabilization. Natural variations between testing environments and testing day/time of day did not affect subject performance (see Supplementary Material: *1. Environment and testing time analysis*).

#### Stimuli

Gaussian noise stimuli were generated using MATLAB (MATLAB R2013a, Version 8.1.0.604) following the procedure described in Zhang et al. (2008). We filled a 480 × 480-pixel matrix with multiple Gaussian blobs of different sizes and contrasts. The positions of the blobs were chosen from a uniform distribution over the image. Three possible blob sizes (standard deviations) were chosen from a bivariate normal distribution. To keep the appearance of the stimuli balanced, the number of blobs were kept inversely proportional to their size (fewer large blobs, more small blobs). Each blob appeared with an intensity (luminance) amplitude chosen randomly from a list of six possible values between − 1 (black) and 1 (white), with mean gray set at 0. These values, in terms of deviation from mean gray, were − 0.3 (− 15%), − 0.25 (− 12.5%), − 0.2 (− 10%), 0.2 (10%), 0.25 (12.5%), and 0.3 (15%). Table S1 (Supplementary Material: *2. Noise parameters*) gives an overview of the size and frequency distribution of blobs within each noise image. An example noise image and its resulting spatial frequency spectrum are shown in Fig. S1a.

**Fig. 1:**
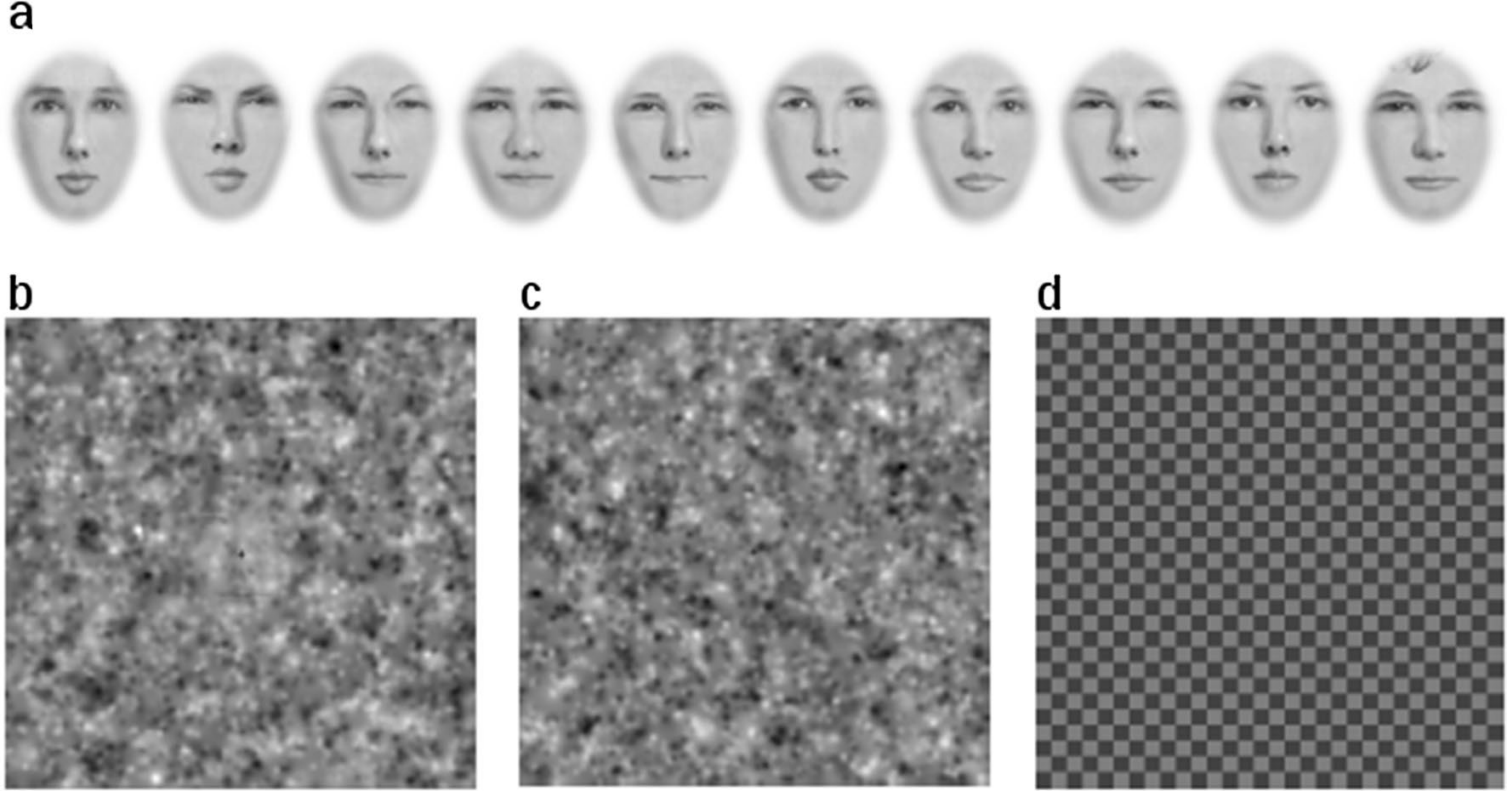
**a** The ten faces viewed during training trials in all experiments, and in the main task of Exp. 2. **b** An example of a face embedded in noise from Face Set 1, used during training trials in Exp. 1. **c** An example of a face embedded in noise from Face Set 2, used during training trials in Exp. 1. **d** The checkerboard image that was used on attention control trials in Exp. 1

Face stimuli (only presented during training) were generated for a different study (Towler, Parketny, & Eimer, 2016) and obtained from the corresponding author. These were ten male faces created with computerized facial composite software (FACES 4.0; IQ Biometrix; https://www.iqbiometrix.com/products_faces_40.html). All features (e.g., eyes, nose, mouth) were unique to each face. For the current study, we additionally removed the hair and facial outline, and blurred the edges of the faces using Photoshop. Images were then scaled to 398 × 398 pixels (~ 11.15° visual angle; see Fig. 1a). From here, we applied an elliptical transparency gradient with a 20-pixel radius around the center of the images to shrink visibility of non-central facial features (Face Set 1; see Fig. 1b). We then performed the same transformation with a 40-pixel radius around central pixels to shrink the faces further (Face Set 2; see Fig. 1c). We presented both transformations of images embedded in the Gaussian noise during the training, so that faces were gradually more difficult to detect, and to remain consistent with Zhang et al. (2008).

#### Procedure

All experimental materials (demographics information, training, main experiment, questionnaires) were programmed in python with the Psychopy psychophysics toolbox (Peirce, 2007) and were completed on the computer. After a demographics form, an instruction screen for the training appeared (full instructions can be found in the Supplementary Material: *3. Experiment instructions*). Subjects were told that faces would appear in the center of a Gaussian noise image about 50% of the time. They were to press “1” if they saw a face, and “2” if they did not see a face, as soon as the image disappeared. Once subjects pressed any key, the training began (6 blocks of 20 trials). All stimuli appeared against a gray background in the center of the screen. The trial structure was as follows: 200 ms fixation cross, 600 ms Gaussian noise image, 1200 ms fixation cross (response period). In the first two blocks of training, a randomly chosen face from Face Set 1 appeared at 40% opacity in the center of the Gaussian noise on half of trials, for the full 600 ms. In the next two blocks of training, a randomly chosen face from Face Set 2 appeared at 20% opacity in the center of the Gaussian noise on half of trials, for the full 600 ms. In the final two blocks of training, no faces appeared, unbeknownst to the subjects.

Following the training, subjects were reminded with written instructions (full instructions in the Supplementary Material: *3. Experiment instructions*) that faces would appear about 50% of the time. Additionally, they were told that if they saw a checkerboard pattern on some trials, they should not respond to it. Subjects were not verbally instructed about the checkerboard images (see Fig. 1d). These images were included to ensure that subjects read the instructions, since one of our hypotheses was that responses were influenced by instructions. Subjects were excluded from analysis if they responded on more than five checkerboard trials in the whole experiment. The main experiment followed the same procedure as the training, but with 4 blocks of 120 Gaussian noise trials (plus 10 checkerboard trials randomly interspersed within each block). Faces never appeared in the main experiment.

Following the main experiment, subjects completed two computerized questionnaires (written versions are presented in Supplementary Material: *4. Questionnaires*). The first questionnaire, designed by the experimenters, required subjects to report on various qualities of the faces that they had seen in the main experiment (Qualities of Faces questionnaire, or QoF). At the end, subjects had the option to write in any additional information about their perceptions. The second questionnaire was the vividness of visual imagery questionnaire (VVIQ; Marks, 1973). Subjects were asked to form visual images of four scenes and subjectively rate (on a 1–5 scale) the imagined vividness of visual details about those scenes. We flipped the scale from the original VVIQ so that a rating of “1” meant no visual image was formed, and a rating of “5” meant that the image was perfectly clear and as vivid as perception.

### Results

Similar to previous studies, we found a high amount of individual variability in the proportion of face-present responses (SD = 17.47%; all group-average results can be found in the Supplementary Material: *5. Group-average results*). Unexpectedly, many subjects chose not to respond on several trials. We hypothesized that this was largely due to greater perceptual uncertainty in subjects who were more pareidolia prone, because we found extremely strong evidence that the number of non-responses was negatively correlated with the number of face-absent responses (see Supplementary Material: *6. Exploring non-responses*). In other words, subjects who were more certain that there was no face (non-pareidolia prone) had fewer non-responses, suggesting increased confidence that no face was present.

Although an interpretation of non-responses can only be speculative, our measure of pareidolia proneness should account for the different number of responses made by subjects: specifically, we wanted to determine the likelihood of an observed number of face-present responses, weighted by the total number of responses and the probability of making a face-present response. This should help to tease apart low numbers of face-present responses due to indecisiveness (more likely if there is a relatively low number of total responses) versus actually not seeing faces (more likely if there is a relatively high number of total responses). For more details concerning the logic of using probabilities over proportions, see Supplementary Material: 7. *Proportions vs. probability*.

To do this, we calculated pareidolia proneness as binomial probabilities using the SciPy scientific computing library in Python (Vertanen et al., 2020). This analysis takes three inputs for each subject: the number of successes (face-present responses), the total number of trials (total responses), and the expected probability of making a face-present response. We input 0.39 as our a priori expected probability, which was the group-average proportion of face-present responses in Exp. 1 of Rieth et al. (2011; Exp. 1 was the same task as ours). Because we wanted to distinguish between individual probabilities that were above and below the expected probability (probabilities above 0.39 indicated higher pareidolia proneness, whereas probabilities lower than 0.39 indicated lower pareidolia proneness), our results are based on one-tailed tests. This resulted in probability values between 0 and 1, with values closer to 1 reflecting higher probability of pareidolia proneness.

**Fig. 2:**
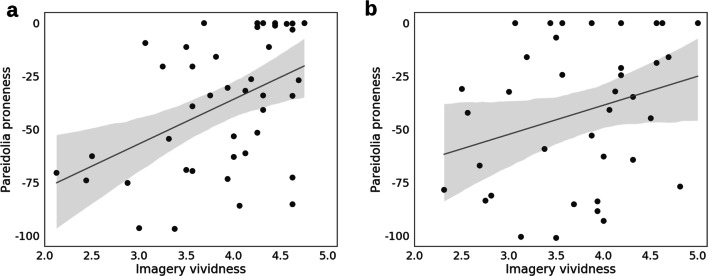
A trend line (gray) and linear regression model (95% CI; gray shading) were fitted to the data for **a** Exp. 1a (*N* = 42); and **b** Exp. 1b (*N* = 37). Imagery vividness (mean rating on the VVIQ) is displayed on the *x*-axis and pareidolia proneness (binomial probability) is shown on the *y*-axis. Probability values were log-transformed for visualization purposes, so values that appear closer to 0 (log-transformed) are probabilities that are closer to 1

Our main analysis of interest was a ranked correlation between pareidolia proneness and imagery vividness (see Fig. 2). The imagery vividness measure was a subject's mean score on the VVIQ (scores ranging between 1 and 5), with larger numbers corresponding to more vivid imagery. The analysis was one-tailed to reflect the hypothesized positive direction of the correlation.

We performed a Kendall’s tau-b (*τ*_**b**_) correlation and calculated the likelihood of the alternative hypothesis using Bayes Factor (BF) analysis in JASP (JASP Team, 2019) with the stretched beta prior width set to 1. This revealed very strong evidence for a positive correlation between pareidolia proneness and imagery vividness (*τ*_**b**_ = 0.321, BF_+0_ = 31.011, 95% credible intervals (CIs) [0.102, 0.497]; see Fig. 2a).

## Experiment 1b: instruction change

Experiment 1a demonstrated a positive association between pareidolia proneness and imagery vividness, but there could be several reasons for this effect. For one, it is possible that subjects who were more uncertain in their percepts felt more encouraged to respond positively sometimes due to suggestive instructions (i.e., “faces will appear about 50% of the time”), rather than actually being prone to see more faces. We were, therefore, interested to investigate the effect of alleviating acquiescence response bias on response profiles. This would allow us to examine the influence of suggestibility on the relationship between imagery vividness and pareidolia proneness.

Experiment 1b was the same as Exp. 1a in terms of design, but subjects were now instructed to respond positively only if they thought they may have actually seen a face, and not to worry if they did not see faces over several trials (for the exact instructions, see Supplementary Material: *3. Experiment Instructions*).

### Methods

#### Subjects

Fifty-one subjects were recruited via the MaXLab online recruitment tool. All data were collected over 3 testing days, and were analyzed following complete data collection. Subjects were reimbursed for 10 euros for the 1-h experiment, as per laboratory guidelines. All subjects provided written, informed consent to take part in the experiment. The experiment was approved by the Otto-von-Guericke University ethics committee and adhered to the tenets of the Declaration of Helsinki. We obtained a diverse sample of bachelor's and master's students studying engineering, business/economics, social science, mathematics, medicine, sport science, computer science, biology, environmental science, and journalism.

13 subjects were excluded due to failing to pass the attention check. We had a final group size of 37. Our sample included 12 women, 4 left-handed and 1 ambidextrous, 16 English speakers, and 21 German speakers, with a mean age of 25.49 years (range 20–37).

#### Apparatus

Experiment 1b took place at the same times and in the same environments as Exp. 1a. There was no general effect of environment on subject performance (see Supplementary Material: *1. Environment analysis*).

#### Stimuli

Stimuli were the same as those used in Exp. 1a.

#### Procedure

The experimental procedure was the same as in Exp. 1a, except instructions were changed for both training and the main experiment (see Supplementary Material: *3. Experiment instructions*). The new instructions emphasized the importance of responding positively only if subjects had the impression that faces actually appeared, rather than responding positively about 50% of the time. This was done to gauge the effects of acquiescence response bias on performance, specifically how the removal of suggestive instructions influences the relationship between pareidolia proneness and imagery vividness. Subjects completed digital versions of the QoF and VVIQ after the main experiment.

### Results

A Bayesian Mann–Whitney *U* test conducted on the number of face-present responses in Exp. 1a versus those made in Exp. 1b revealed anecdotal evidence that changing instructions reduced acquiescence (*U* = 581.500, BF_10_ = 1.731; full group-average results are reported in the Supplementary Material: *5. Group-average results*).

As in Exp. 1a, there was a high number of non-responses. These again seem to reflect uncertainty, since we found extremely strong evidence for a negative correlation between the number of non-responses and face-absent responses (see Supplementary Material: *6. Exploring non-responses*). We again calculated pareidolia proneness as one-tailed binomial probabilities, with the expected probability set to 0.39. A one-tailed Kendall’s *τ*_**b**_ correlation and BF analysis conducted in JASP revealed anecdotal evidence for a positive correlation between pareidolia proneness and imagery vividness (*τ*_**b**_ = 0.206, BF_+0_ = 1.940, 95% CIs [0.024, 0.405]). This weaker evidence for a relationship compared to Exp. 1a suggests that there is at least some influence of suggestibility on responses.

Additional qualitative analyses performed on the data from Exp. 1a and Exp. 1b can be found in the Supplementary Material: *8. Qualitative results*, Fig. S2, and Table S2.

## Interim analyses

### Perceptual sensitivity and response bias

The results of Exp. 1a and Exp. 1b led us to probe deeper into possible effects of perceptual sensitivity and response bias on pareidolia proneness in the main experiment. For this, we looked to the training data: these data contained actual faces in the first four blocks of trials, so d-prime (*d*ʹ) and criterion (*c*) could be calculated and correlated with main-experiment responses. Specifically, we calculated training-phase *d*ʹ and *c* for the 40% contrast face detection blocks and the 20% contrast face detection blocks separately. This resulted in four, two-tailed Bayesian *τ*_**b**_ correlations with main-experiment pareidolia proneness, performed separately for Exp. 1a and Exp. 1b.

For both Exp. 1a and Exp. 1b, we found anecdotal evidence for a correlation between training-phase *c* for 40% contrast faces and main-experiment pareidolia proneness (Exp. 1a: *τ*_**b**_ = 0.231, BF_10_ = 1.811, 95% CIs = [0.014, 0.417]; Exp. 1b: *τ*_**b**_ = 0.188, BF_10_ = 0.785, 95% CIs = [− 0.036, 0.387]). This suggests a slight bias for more pareidolia-prone subjects to report the presence of faces in these blocks. However, when faces became more difficult to see (20% contrast blocks), we found moderate evidence for a true null relationship between *c* and pareidolia proneness in both Exp. 1a (*τ*_**b**_ = − 0.072, BF_01_ = 3.986, 95% CIs = [− 0.269, 0.134]) and Exp. 1b (*τ*_**b**_ = − 0.005, BF_01_ = 4.700, 95% CIs = [− 0.214, 0.208]). This suggests that subjects changed their response strategies when faces became more difficult to detect, and pareidolia-prone subjects were no differently biased than non-pareidolia-prone subjects.

We further found moderate evidence for a true null relationship between *d*ʹ for 40% contrast face detection and pareidolia proneness in both Exp. 1a (*τ*_**b**_ = 0.075, BF_01_ = 3.920, 95% CIs = [− 0.132, 0.271]) and Exp. 1b (*τ*_**b**_ = 0.087, BF_01_ = 3.552, 95% CIs = [− 0.132, 0.291]). This suggests that subjects generally showed similar signal-to-noise discrimination ability in these training blocks. For the correlation between training-phase *d*ʹ for 20% contrast faces and main-experiment pareidolia proneness, we found anecdotal evidence for a true null relationship for Exp. 1a (*τ*_**b**_ = 0.181, BF_01_ = 1.271, 95% CIs = [− 0.032, 0.369]) and moderate evidence for a true null relationship for Exp. 1b (*τ*_**b**_ = − 0.082, BF_01_ = 3.679, 95% CIs = [− 0.289, 0.134]). Together, the results suggest no relationship between perceptual sensitivity or response bias (on training trials) and pareidolia proneness (in the main experiments).

### Behavioral classification analyses

From the previous analyses, we can rule out perceptual sensitivity and response bias as possible explanations for the patterns of responses seen in Exp. 1a and Exp. 1b. Next, we were interested to find out whether some people are simply more likely than others to pick up on subtle face-like patterns that may appear randomly in the noise images. Specifically, it is possible that the random distribution of Gaussian blobs in the noise images happened to contain face-like patterns, which could have influenced face-present responses in subjects with a lower “signal detection” decision criterion (Dolgov & McBeath, 2005). To further explore this, we conducted classification analyses on the correlation between subject responses and low-level properties of the Gaussian noise images used in these experiments. Previous studies have used similar methods to “render” the shapes of expected objects in pure noise by correlating the observed noise patterns with subject responses (“present” vs. “absent”; Gosselin & Schyns, 2003). In our first experiment (Exp. 1a, Exp. 1b), the identity of each noise image was recorded whenever subjects made a response following a static image presentation. The correlation between responses on these images and low-level noise properties were then analyzed for Exp. 1a and Exp. 1b separately (due to face-presence response differences).

First, we conducted within-subjects reverse correlation analyses as described by Rieth et al. (2011). Here we computed a Classification Image (CI) for each subject. For this, the grayscale values of all pixels were correlated with face-present and face-absent responses over all 480 trials. The CI was then divided into nine ROIs of equal size (defined by overlaying a 3 × 3 grid over each noise image), to determine if results were tied to specific locations in the image. The median correlation for each ROI (including the whole-image ROI) was computed for each participant, then analyzed using an ANOVA with subject as a random factor (see Supplementary Material: *9. Bottom-up analyses*, Fig. S3). This revealed no significant differences for either Exp. 1a (*F*(1, 369) = 0.61, Pr(> *F*) = 0.77) or Exp. 1b (*F*(1, 288) = 0.371, Pr(> *F*) = 0.94; see Supplementary Material: *9. Bottom-up analyses*, Table S4 for a summary of the *t* tests performed within each ROI). Rieth and colleagues concentrated their analyses to the center part of the images (where correlations were highest), corresponding roughly to locations where actual faces appeared on training trials. The authors found differences between the median correlations of the left part of the center region compared to the center-right. We, therefore, split our central region into two halves to analyze this potential difference. This analysis yielded no left–right differences for either Exp. 1a (*t*(41) =  − 0.59, *p* = 0.55) or Exp. 1b (*t*(36) =  − 0.25, *p* = 0.80).

We then performed a between-subjects classification analysis, also as described by Rieth et al. (2011), to determine any meaningful clusters of pixel contrasts in images that elicited face-present responses. For this, we computed the proportion of face-present responses for each noise image (face-present responses divided by the sum of face-present and face-absent responses), then correlated this with the grayscale value of each pixel over all 480 images.

**Fig. 3:**
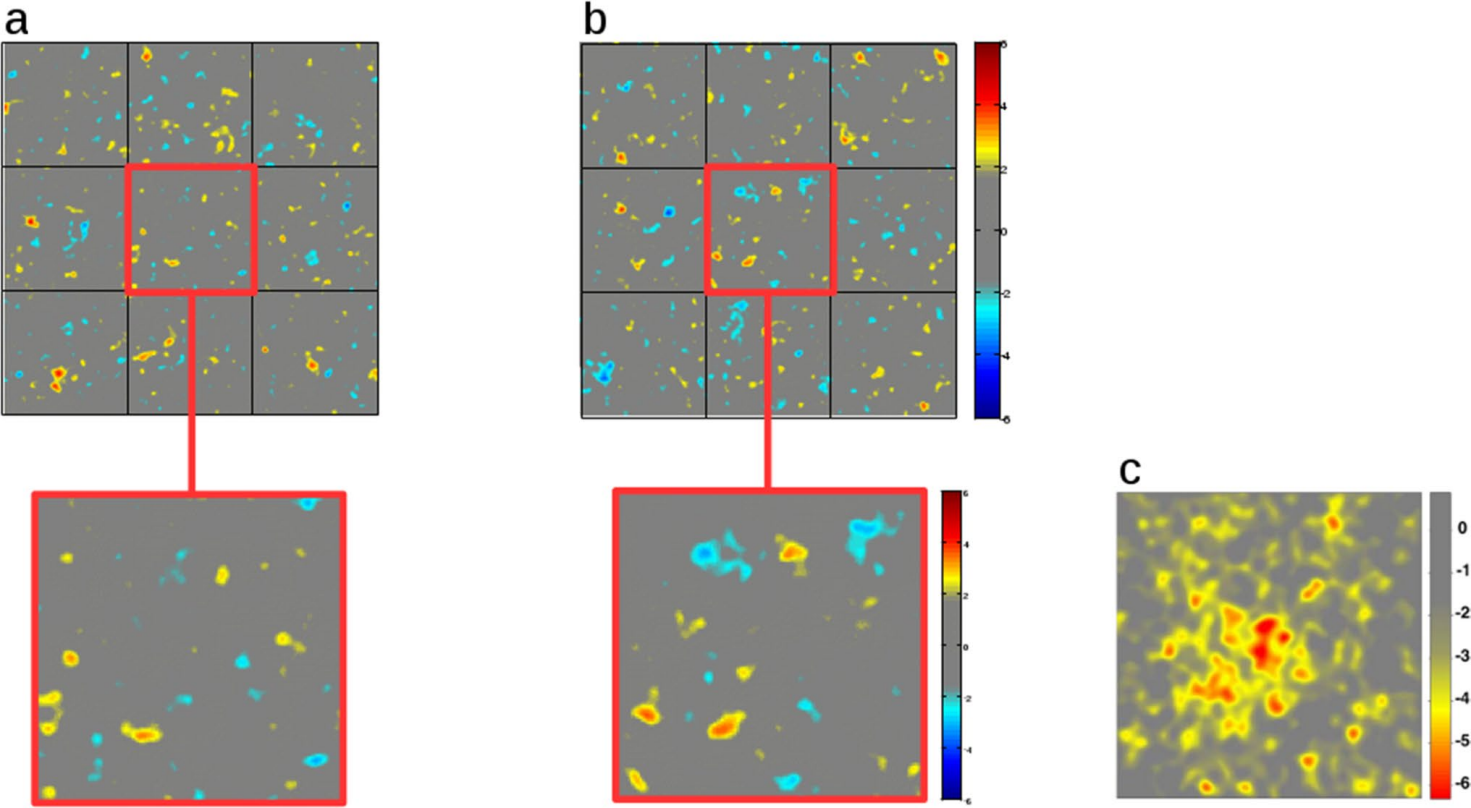
**a**, **b** Yellow-to-red colors indicate *z* scores denoting positive correlations between subject responses and pixel grayscale values, and light blue-to-dark blue colors indicate *z* scores denoting negative correlations for Exp. 1a (**a**) and Exp. 1b (**b**). The range of z scores shown in the figure was set to match that of Rieth et al. To compare our results to those of Rieth and colleagues, we zoomed in on the central grid location where the authors of the previous study performed their analyses, and compared the (non-significant) clusters contained in our CIs to theirs (**c**). This panel is reproduced, on a Creative Commons license, from Fig. 3 in Rieth, C. A., Lee, K., Lui, J., Tian, J. & Huber, D. E. *Faces in the Mist: Illusory Face and Letter Detection* in *i-Perception*, dx.doi.org/10.1068/i0421, volume 2, pages 458–476, 2011, SAGE Publications Inc

We performed 5000 Monte Carlo simulations to find the expected SDs and mean correlations for random responses. For each Monte Carlo simulation, the order of the 480 face-present response proportions was randomly shuffled and correlated with the grayscale value of all pixels over all 480 images. Means and SDs were calculated for each pixel, and we used these to compute *z* scores for each pixel of the experimental CI (see Supplementary Material: *9. Bottom-up analyses* Table S5 for a summary of these results). As in the earlier study, we adjusted the *z* score threshold to correct for multiple comparisons using Šidák correction with *α* set to 0.05. In our study, this resulted in a threshold of *z* =  ± 4.259, which revealed no significant clusters in any part of the image. Figure 3a, b shows the resulting CIs for Exp. 1a and Exp. 1b. Each CI was further divided into nine ROIs as in the within-subjects analysis, to determine if any (non-significant) clusters were tied to specific locations. While Rieth et al. found face-like clusters in the center region (particularly the left side), we did not find any meaningful clustering in our noise images to any comparable extent (see Fig. 3c).

We conducted two more types of analyses on the noise images to more comprehensively explore potential bottom-up influences on responses. These included analyses of the difference in luminance and Root Mean Square Contrast (CRMS) between the means of images that elicited face-present and face-absent responses in Exp. 1a and Exp. 1b. Furthermore, subjects were median split into a “high” or “low” imagery vividness group to determine if individuals with different imagery vividness relied on different bottom-up strategies. In that case, we should at least find physical differences between images that elicited face-present and face-absent responses in the group with more vivid imagery. However, regardless of the type of analysis performed (luminance, CRMS), location analyzed (whole-image, ROI), experiment (1a, 1b), or group split (low-imagery, high-imagery), we found no low-level differences between images that elicited face-present and face-absent responses (see Supplementary Material: *9. Bottom-up analyses*, Fig.S4, and Table S6). For additional analyses of the Gaussian noise images based on the profile of responses in Exp. 1, see Supplementary Material: *9. Bottom-up analyses.*

## Interim discussion

The relationship between pareidolia proneness and imagery vividness detailed in Exp. 1 cannot be explained by perceptual sensitivity, response bias, or face-like patterns in the noise images. Reducing acquiescence response bias weakens this relationship, which could indicate some influence of suggestibility on responses. One puzzling finding that we could not control was that many subjects chose not to respond on several trials. Since it was a replication, Exp. 1 was not programmed to ensure that subjects responded on each trial (there was a fixed 1200 ms response period that terminated whether subjects made a response or not, as in previous studies). An investigation of non-responses (see Supplementary Material: *6. Exploring non-responses*) led us to hypothesize that these might be meaningful, and perhaps related to a lack of confidence in percepts. One alternative explanation is that non-responses reflect a lack of motivation due to no real faces being present during the main experiment. For Exp. 2, it was, therefore, important to change the design to address our hypothesis directly.

To follow-up on our “uncertainty” hypothesis, we created a new task to increase perceptual ambiguity. Previous studies suggest that various illusory experiences, such as depth and geometric patterns, can emerge in dynamic displays of random noise (Fiorentini & Mackay, 1965; MacKay, 1965). We hypothesized that if simple illusions can seem to appear in dynamic displays, then these displays might also be conducive to complex illusions, such as face pareidolia. We, therefore, used dynamic Gaussian noise in our next pareidolia experiment. An initial pilot experiment (see Supplementary Material: *10. Pilot experiment A: Short dynamic noise*) revealed that 3 s of dynamic Gaussian noise greatly increased the proportion of face-absent responses across all subjects (mean = 88.52%, SD = 15.05%), to the extent that there was now very little variability in response profiles and virtually no relationship between pareidolia proneness and imagery vividness. We, therefore, created an entirely new design that recruited continuous dynamic noise for an entire run (~ 5.5 min) to boost illusory experiences. This also abolished any trial structure, removed a need for face-absent responses (which dominated responses in Exp. 1, and even more so in Pilot Exp. A), and further reduced response pressure.

Next, we presented real faces at perceptual threshold infrequently throughout the experiment to sustain subject motivation, due to the task requiring relatively few responses. Importantly, we now collected confidence ratings after every face-present response, to increase the likelihood of subjects making a response even when they were unsure about the presence of a face. This addition allowed us to directly measure confidence, rather than indirectly inferring it from non-responses.

Our final paradigmatic change was to extend the size of the noise images to fill the entire display. Our qualitative analyses from Exp. 1 revealed that people who reported the most faces also saw larger faces in variable locations, and details that were neither related to low-level noise patterns or the faces presented on training trials (e.g., “Einstein’s face” or “ape faces”, see Supplementary Material: *8. Qualitative Results*). We, therefore, hypothesized that increasing the size of the noise would also enhance this variability in pareidolia experiences. Importantly, this change helps to address a potential confound: in the previous experiment, noise image size was based on pixel values and we did not stabilize subjects’ heads. Because illusory experience is different across the visual field (see Eagleman, 2001 for a review), it could be argued that subjects who saw more faces happened to be at a critical viewing distance for pareidolia experiences. Extending the noise to the majority of the visual field, therefore, controls for this. In addition to extending the noise, we stabilized subjects’ viewing distance for the next experiment.

In addition to the questionnaires from Exp. 1 (VVIQ, QoF), we included the 20-Item Prosopagnosia Index (PI20; Shah et al., 2015) for this experiment. Prosopagnosia is the selective impairment of face recognition, and it is highly related to low-imagery vividness (Grüter et al., 2009). It is possible that the relationship between pareidolia proneness and imagery vividness is driven by individual differences in prosopagnosia. We, therefore, also conducted correlations between imagery vividness, pareidolia proneness, and scores on the PI20.

## Experiment 2a: continuous dynamic Gaussian noise

### Methods

Our goal with this experiment was to better understand the relationship between pareidolia proneness and imagery vividness by boosting perceptual ambiguity; eliminating response pressure; and controlling for visual field confounds, motivation, and the possibility that prosopagnosia may drive the effects. We also directly measured subjective confidence in percepts to explore the role of confidence in pareidolia proneness.

#### Subjects

The experiment was conducted at the Institute of Psychology at Otto-von-Guericke University in Magdeburg, Germany. 30 students and faculty were recruited and performed the experiment for course credit (students), or purely in the interest of science (faculty). All subjects provided written, informed consent to take part in the experiment. The experiment was approved by the Otto-von-Guericke University ethics committee and adhered to the tenets of the Declaration of Helsinki. Our sample included 23 women, with a mean age of 23.53 years (range 19–34). Although our sample was dominantly composed of women, a gender-split analysis of the results of Exp. 1a and Exp. 1b suggest no gender differences in pareidolia proneness or imagery vividness for either experiment (see Supplementary Material: *11. Gender analyses*).

#### Apparatus

The computer monitor was a 24-inch Samsung with 1920 × 1080 screen resolution and 60 Hz refresh frequency. There were 65 cm between the monitor and the table edge. Subjects' heads were stabilized with a combined head and chin rest mounted to the table. Subjects performed the experiment within a soundproof booth and in complete darkness.

#### Stimuli

A new set of 480 Gaussian noise stimuli were generated to fit the dimensions of the entire screen (1920 × 1080 pixels). Aside from the different size dimensions, stimuli were created in the same way as in the previous experiments. Face stimuli (presented during training and the main experiment) were the full-face versions (see Fig. 1a) of the same ten images used in the previous experiments. Faces were always presented at 19% of full contrast. Faces were initially presented at 25% opacity during training trials, but this value changed according to individual perceptual threshold and was updated after every experimental block (see Supplementary Material: *12. Perceptual threshold calculation* for details).

#### Procedure

Subjects first filled out a consent form and a paper version of the VVIQ. They then entered the soundproof booth and both chair and chin rest were adjusted so that they were positioned with their gaze centered on the screen. Subjects were first shown an example of the dynamic noise presented in the experiment. They were told that the experiment would involve looking at this noise continuously for about five and a half minutes. After each block, they were allowed to take a break if needed. The experimenter provided verbal instructions about the task (see Supplementary Material: *3. Experiment instructions*). Subjects were told that faces would be difficult to see. They were encouraged to keep their minds open for various locations, sizes, frequencies, and contrasts of the faces, and to respond even if they were not confident in their perception, because they would be able to make a confidence rating (on a 1–4 scale) about what they saw. A confidence rating of “1” meant the subject pressed the key by accident, whereas a confidence rating of “4” meant they distinctly saw a face. They were then allowed to ask questions, but the experimenter was explicitly told not to provide any hints about what to expect about the faces. Subjects were simply told to keep their minds open for anything.

Following the instructions, subjects completed four training blocks. Each block contained 20 “trials” of 4 s, with each trial composed of 60 randomly selected Gaussian noise images presented sequentially for ~ 67 ms each (15 Hz; Hz). Because each trial was a continuous presentation of noise images, subjects were unaware of any trial structure. During each block of the training, faces appeared on 15 randomly selected trials for a duration of 500 ms. They could appear at a distance of either 5 or 7° of visual angle from fixation, in any quadrant of the screen. Faces could appear at 6, 7, 8, or 9° of visual angle in size. One face appeared on a single trial, and its identity was chosen randomly from the ten images. Subjects had 2 s to respond to the presence of a face, or else the response was counted as a miss for perceptual threshold calculation.

After the training, if subjects had no further questions, they began the main experiment. The main experiment was composed of 7 blocks of 80 “trials” of 4 s, following the same noise specifications as in the training. During each block of the main experiment, faces appeared on 20 pseudo-randomly selected trials for a duration of 500 ms: faces could not appear in two sequential trials, and they could not be absent for more than 7 sequential trials. Within these parameters, the 20 face-present trials were chosen randomly. This ensured variability in the time between face presentations. Whenever subjects responded with the “1” key (which measured face-present responses), a confidence screen appeared for 2 s. Subjects were required to indicate their confidence in their response on a 1–4 scale. The confidence screen remained on-screen for 2 s regardless of response speed, after which the trials continued. The confidence screen did not cut into trial time, so more responses corresponded to a longer experiment time. The experiment took no longer than 50 min. Following the main experiment, subjects completed paper versions of the QoF and the PI20.

#### Analyses

A detection response was coded if the “1” key was pressed within 2 s of the onset of a face. If subjects made a detection response more than 2 s after face onset, the response was not recorded for that trial. A misperception response was coded if the “1” key was pressed at any time during a face-absent trial. Any response with a confidence rating of “1” (mistaken key press) was not coded as either a detection or a misperception, but was nevertheless recorded to determine the number of impulsive responses. We determined that if subjects made a response but did not provide a confidence rating within the 2-s time window, the trial would be discarded—however, all subjects in the experiment were able to provide confidence ratings for every response.

We could not compare face-present and face-absent responses in this experiment, and we could no longer quantify non-responses. We, therefore, performed analyses on the total number of detection and misperception responses, as well as subjects' average confidence ratings for each response condition, separately. As before, our main analysis of interest was a correlation analysis between pareidolia proneness (here measured as the total number of misperceptions) and imagery vividness (mean VVIQ-score).

### Results

There was quite some individual variability in the total number of misperceptions (SD = 28.37; also see Supplementary Material: *5. Group-average results*). Because confidence ratings were dependent on subjects making responses, subjects who made 0 misperception responses (and therefore, gave no misperception confidence ratings) were excluded from correlation analyses (*N* = 5). We had a final sample size of 25 for correlations.

**Fig. 4:**
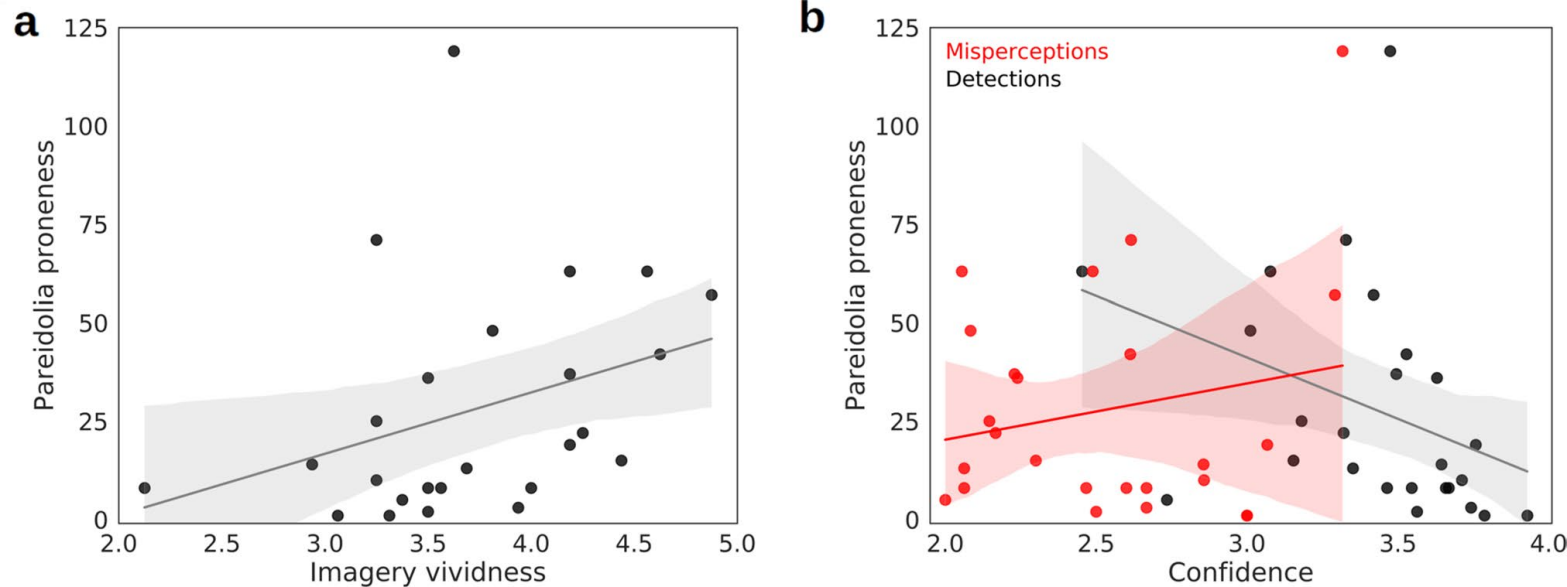
**a** A trend line (gray) and linear regression model (95% CI; gray shading) were fitted to the data of Exp. 2a (*N* = 25). The scatterplot shows the correlation between pareidolia proneness (number of misperceptions; *y*-axis) and imagery vividness (mean rating on the VVIQ; *x*-axis). **b** Two trend lines and linear regression models (95% CIs) were fitted to the data of Exp. 2a (*N* = 25) for correlations between pareidolia proneness (*y*-axis) and confidence (4 = high certainty; *x*-axis). The correlation with detection confidence is shown in grayscale, and the correlation with misperception confidence is shown in shades of red

A one-tailed, Bayesian Kendall’s *τ*_**b**_ correlation analysis, conducted on the relationship between pareidolia proneness and imagery vividness, revealed moderate evidence for a positive correlation (*τ*_**b**_  = 0.333, BF_+0_ = 6.793, 95% CIs [0.062, 0.549]; see Fig. 4a). Comparatively, we found no evidence for a correlation between real face detections and imagery vividness in a two-tailed test (*τ*_**b**_  = − 0.183, BF_10_ = 0.564, 95% CIs [− 0.419, 0.090]).

Next, we conducted correlation analyses between pareidolia proneness and confidence ratings for both detections and misperceptions. We hypothesized that the relationship would be negative (less confidence in perception is associated with higher pareidolia proneness). A one-tailed, Bayesian Kendall’s *τ*_**b**_ correlation analysis, conducted on the relationship between pareidolia proneness and detection confidence, revealed very strong evidence for a negative correlation (*τ*_**b**_  = − 0.426, BF_−0_ = 35.553, 95% CIs [− 0.629, − 0.128]; see Fig. 4b). There was no evidence for a correlation between pareidolia proneness and misperception confidence (*τ*_**b**_  = − 0.037, BF_−0_ = 0.319, 95% CIs [− 0.006, 0.317]; see Fig. 4b). We instead found moderate evidence for a true null relationship (BF_0−_ = 3.137). This suggests that pareidolia proneness in our experiment was related to a lack of confidence in real, rather than illusory, percepts. It is possible that the observed relationship between pareidolia proneness and confidence was simply a consequence of perceptual thresholding: subjects who had a relatively lower face detection threshold may have been more confident in their responses because the faces were easier to see for these subjects. To rule this out, we conducted a two-tailed correlation analysis to determine the relationship between the final opacity value of real faces for each subject and their face detection confidence. This revealed no evidence for a correlation (*τ*_**b**_  = 0.035, BF_10_ = 0.264, 95% CIs [− 0.226, 0.285]), rather showing moderate evidence for a true null relationship (BF_01_ = 3.786).

For completeness, we also explored correlations between imagery vividness and perceptual confidence in two-tailed tests. We found anecdotal evidence for a negative correlation with detection confidence (*τ*_**b**_  = − 0.261, BF_10_ = 1.262, 95% CIs [− 0.487, 0.022]). We found no evidence for a correlation with misperception confidence (*τ*_**b**_  = − 0.031, BF_10_ = 0.262, 95% CIs [− 0.228, 0.283]), and instead found moderate evidence for a true null relationship (BF_01_ = 3.810).

We then wanted to measure whether motivation differences contributed to pareidolia proneness. Our idea was that subjects who saw more real faces in the noise may have been more motivated to respond that they saw faces when faces were not actually present. We performed a two-tailed Bayesian correlation between the number of real detections and misperceptions, and found no evidence for a relationship (*τ*_**b**_ = − 0.227, BF_10_ = 0.857, 95% CIs [− 0.457, 0.052]).

Finally, we performed two-tailed Bayesian correlations on the relationship between prosopagnosia, pareidolia proneness, and imagery vividness. This revealed no evidence of a relationship with prosopagnosia (pareidolia proneness: *τ*_**b**_ = − 0.110, BF_10_ = 0.340, 95% CIs [− 0.355, 0.0156]; imagery vividness: *τ*_**b**_ = − 0.192, BF_10_ = 0.610, 95% CIs [− 0.427, 0.082]).

## Experiment 2b: continuous dynamic white noise

Experiment 2a demonstrated that continuous dynamic Gaussian noise can elicit the experience of face pareidolia in subjects with vivid imagery. Importantly, our design changes from the previous experiment demonstrated that the relationship between pareidolia proneness and imagery vividness is not dependent on a static presentation of noise, response pressure, or changes in viewing distance or noise size. Because of the high number of non-responses in Exp. 1, we also directly explored confidence in percepts in Exp. 2a. These results revealed a strong negative relationship between pareidolia proneness and confidence in real percepts that cannot be explained by motivation differences. We hypothesized that pareidolia-prone subjects show lower confidence in percepts due to a weaker ability to tell the difference between imagined and real percepts when looking for stimuli in perceptually ambiguous environments. We were, therefore, interested to find out how changing the ambiguity of the environment (by decreasing the amount of noise) affects pareidolia proneness and confidence in responses. Exp. 2b used the same flexible design as Exp. 2a with one important change: Gaussian noise images were replaced with white noise images, which created an objectively more uniform sensory environment (see Supplementary Material: *2. Noise parameters*).

### Methods

#### Subjects

The experiment was conducted at the Institute of Psychology at Otto-von-Guericke University in Magdeburg, Germany. 28 students and faculty were recruited and performed the experiment for course credit (students) or purely in the interest of science (faculty). All subjects provided written, informed consent to take part in the experiment. The experiment was approved by the Otto-von-Guericke University ethics committee and adhered to the tenets of the Declaration of Helsinki. Our sample included 16 women, with a mean age of 27.43 years (range 19–39).

#### Apparatus

The experiment environment was the same as in Exp. 2a.

#### Stimuli

White noise stimuli were 100 screen shots from free stock video footage of TV static white noise found online. An example noise image and its resulting spatial frequency spectrum are shown in the Supplementary Material: *2. Noise parameters*, Fig. S1b. These were the same size dimensions as the Gaussian noise in Exp. 2a so that they filled the entire display. Face stimuli were the same as in Exp. 2a. In this experiment, faces were always presented at 19% of full contrast and 25% opacity. During piloting, subjects recruited a strategy in which any small change in the contrast of the white noise was reported as a face (presumably due to the uniformity of the environment), resulting in a lower face detection threshold that was not based on having seen any face-like features. We, therefore, did not tie real stimulus opacity values to subjective thresholds in this experiment, instead preferring to use the starting opacity values from Exp. 2a.

#### Procedure

Subjects first filled out a paper consent form and the VVIQ, and were given the same task instructions as in Exp. 2a. They completed 4 blocks of training trials like in Exp. 2a, except perceptual threshold was not calculated. Following the training, subjects were asked if they had any questions before beginning the main experiment. The main experiment procedure was the same as in Exp. 2a. At the end of the experiment, subjects filled out the QoF.

### Results

As in Exp. 2a, we found a high amount of individual variability in the number of misperceptions reported (SD = 23.04). The design changes further did not affect the mean number of misperception responses compared to Exp. 2a (*U* = 409.000, BF_10_ = 0.285; see Supplementary Material: *5. Group-average results* for the full analyses). Subjects with 0 misperception responses were removed from analysis in the same manner as in Exp. 2a, which gave us a final sample size of 23 for Bayesian Kendall’s *τ*_**b**_ correlation analyses. Because we could not predict how changing the ambiguity of the environment would change responses and confidence, all tests were two tailed.

**Fig. 5:**
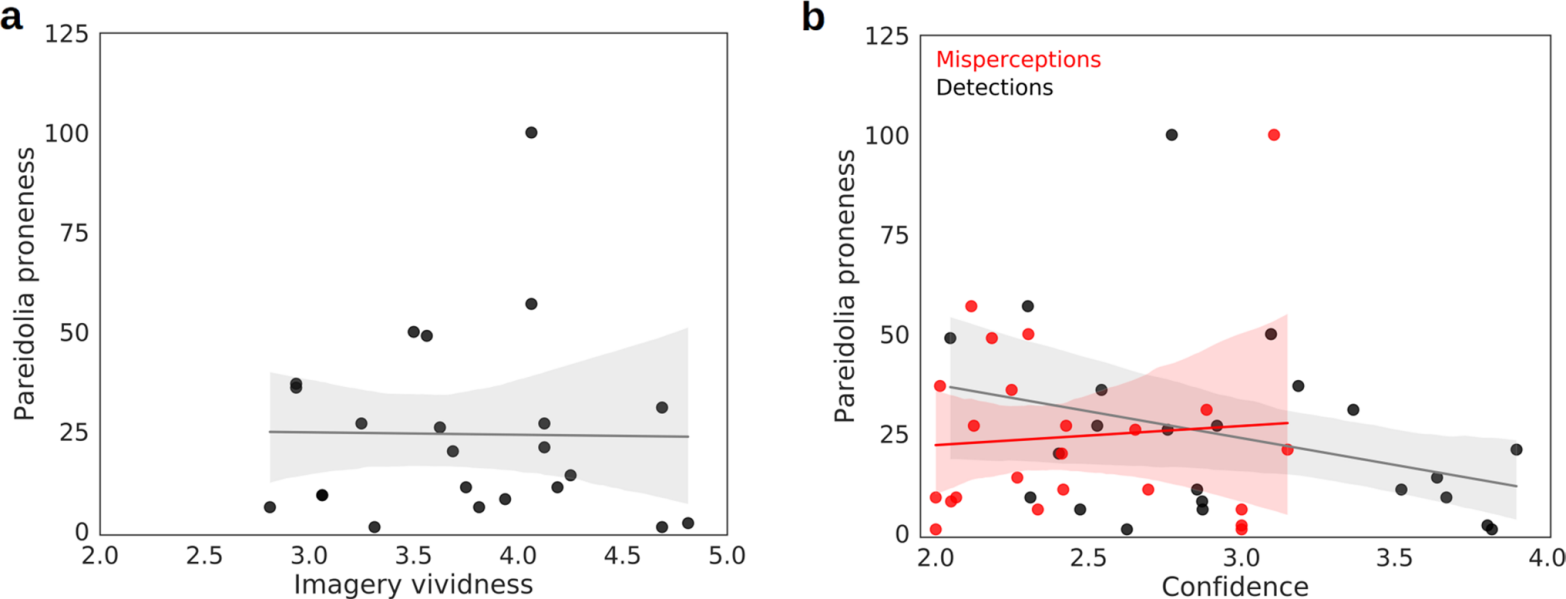
**a** A trend line (gray) and linear regression model (95% CI; gray shading) were fitted to the data of Exp. 2b (*N* = 23). The scatterplot shows the correlation between pareidolia proneness (number of misperceptions; *y*-axis) and imagery vividness (mean rating on the VVIQ; *x*-axis). **b** Two trend lines and linear regression models (95% CIs) were fitted to the data of Exp. 2b (*N* = 23) for correlations between pareidolia proneness (*y*-axis) and confidence (4 = high certainty; *x*-axis). The correlation with detection confidence is shown in grayscale, and the correlation with misperception confidence is shown in shades of red

First, we found no evidence for a relationship between pareidolia proneness (total number of misperceptions) and imagery vividness (*τ*_**b**_ = − 0.081, BF_10_ = 0.307, 95% CIs = [− 0.339, 0.194]), rather finding moderate evidence for a true null relationship (BF_01_ = 3.259). We now found moderate evidence for a positive correlation between detection responses and imagery vividness (*τ*_**b**_ = 0.380, BF_10_ = 5.707, 95% CIs = [0.068, 0.595]) (Fig. 5).

Next, we found no evidence for a correlation between detection confidence and pareidolia proneness (*τ*_**b**_ = − 0.208, BF_+0_ = 0.667, 95% CIs = [− 0.449, 0.082]), rather finding anecdotal evidence for a true null relationship (BF_01_ = 1.498). We furthermore found no evidence for a relationship between misperception confidence and pareidolia proneness (*τ*_**b**_ = − 0.032, BF_10_ = 0.273, 95% CIs = [− 0.295, 0.236]), rather finding moderate evidence for a true null relationship (BF_01_ = 3.661).

For completeness, we then performed correlations between imagery vividness and confidence responses. Now, we found strong evidence for a positive correlation with detection confidence (*τ*_**b**_ = 0.415, BF_10_ = 10.305, 95% CIs = [0.100, 0.625]), and moderate evidence for a positive correlation with misperception confidence (*τ*_**b**_ = 0.402, BF_10_ = 8.265, 95% CIs = [0.088, 0.613]). These results suggest a much stronger relationship between imagery vividness and confidence in Exp. 2b than in Exp. 2a, both of which seem to have a negligent association with pareidolia proneness in Exp. 2b.

We then wanted to measure whether motivation differences contributed to pareidolia proneness. We performed a two-tailed Bayesian correlation between the number of real detections and misperceptions, and found no evidence for a relationship (*τ*_**b**_ = − 0.036, BF_10_ = 0.275, 95% CIs [− 0.299, 0.232]), rather finding moderate evidence for a true null relationship (BF_01_ = 3.641).

## General discussion

In this study, we demonstrated that imagery vividness contributes to pareidolia proneness in perceptually ambiguous environments. This relationship may be influenced by suggestive instructions (Exp. 1b), but cannot be explained by response bias, perceptual sensitivity, or bottom-up patterns in noise (Interim analyses). It furthermore cannot be explained by response pressure, viewing distance, noise size, motivation, prosopagnosia, or gender differences (Exp. 2a). The relationship persists when viewing both static (Exp. 1a) and continuous dynamic Gaussian noise (Exp. 2a). The relationship is abolished when looking for faces in a relatively uniform sensory environment (Exp. 2b). Previous studies of face pareidolia in noise have focused on the paradigm of Exp. 1a (Li et al., 2009, 2010; Liu et al., 2014; Rieth et al., 2011; Zhang et al., 2008; Zimmermann et al., 2019), but our additional experiments demonstrate that this paradigm can be adapted to reveal the various factors that contribute to pareidolia proneness.

We additionally explored the relationship between pareidolia proneness and confidence in percepts, following an unexpected finding in Exp. 1. Our investigation revealed that a lack of confidence in real percepts is associated with pareidolia experiences in relatively noisy (Exp. 2a) but not relatively uniform (Exp. 2b) sensory environments. Altogether, our results suggest that pareidolia experiences are linked to both perceptual uncertainty and visual imagery vividness.

### Pareidolia proneness and imagery vividness

Our main hypothesis for this study was that more vivid imagery contributes to stronger sensory simulations and a greater reliance on imagery for perception. If the boundary between imagination and reality is narrower in people with more vivid imagery, then they should be more prone to anomalous perceptual experiences in a perceptually ambiguous environment. We demonstrated evidence for this hypothesis with Exp. 2; the relationship between imagery vividness and pareidolia proneness persisted in the continuous dynamic Gaussian noise condition (Exp. 2a), but was abolished in the continuous white noise condition (Exp. 2b), in which the main design difference from Exp. 2a was the presentation of more visually uniform sensory input. Combined with the result that the number of misperceptions was statistically similar across Exp. 2a and Exp. 2b (see Supplementary Material, *Group-average results*), we have interpreted the pattern of results as a difference in misperception decision strategy. In the low-contrast environment (Exp. 2b), people could have relied on physical clues to detect illusory faces in the noise (e.g., a cluster of relatively dark pixels may have been misinterpreted as a face), whereas in the high-contrast environment (Exp. 2a), people could not use this strategy, because there were always clusters of dark pixels (a constant feature of the Gaussian noise). In this latter case, people had to rely more on their imagination to detect illusory faces rather than physical clues. Both strategies could elicit a similar number of face-present responses across subjects, but the strategy that relies more on imagery would correlate with imagery vividness.

### Potential side effects of dynamic noise

Interestingly, our correlation between pareidolia proneness and imagery vividness holds in both static and dynamic noise displays. Dynamic visual noise (DVN) has been used in previous studies to try to disrupt imagery, with varying success (Andrade et al., 2002; Avons & Sestieri, 2005; Quinn & McConnell, 1996; Santana et al., 2013). The idea is that bombarding the visual system with irrelevant noise can potentially interfere with image formation and/or retention. One study demonstrated that interference only occurs when the DVN contains task-relevant features (Borst et al., 2012). In our study, the DVN occasionally contained task-relevant features (faint real faces), but this did not cause interference with imagery—at least, the relationship between imagery vividness and pareidolia proneness persisted in our design. This could be because our design was different from other DVN studies in two major ways:

First, people were not instructed to remember a specific image with a given identity, size, or location (as would typically be the case in a visual working memory task); they were rather instructed to look for any face, which could appear in any size or location on the screen. This would allow individuals to activate a flexible, personal representation of a face. If subjects are allowed to create their own image (rather than provided an imposed image), the sensory representation may be altered and optimized over time, making it resistant to interference. Second, our DVN persisted for several minutes, which is much longer than the noise presented in previous studies (a few seconds, at most). Presenting DVN at a critical time window (during image formation, for example) may enhance interference effects (Santana et al., 2013), but subjects in our experiment had as much time as they wanted to form and retain an image. Although we do not know exactly how long DVN must persist for this “resistance-to-interference” effect to occur, it would be interesting for future studies using DVN to investigate these more fine-grained temporal aspects.

Another temporal effect of the DVN is related to the rhythmic frequency of the stimulation. To create our dynamic environments (Pilot Exp.A, Exp. 2), noise images flipped at a frequency of 15 Hz. This frequency was chosen for trivial reasons (the refresh rate of the computers used for experimentation was 60 Hz, and 15 Hz constituted an easily divisible number of frames). Incidentally, several studies have found that visual flicker can elicit visual illusions, which have been reported across nearly the entire range of perceivable frequencies from delta to gamma (1 to ~ 50 Hz; Allefeld et al., 2011; Becker & Elliott, 2006). The strongest illusory experiences, however, are reported to occur in the range of 13.1–16 Hz (Sumich et al., 2018). These illusions occurred infrequently over continuous stimulation periods of at least 30 s. Another study found that it took, on average, over 8 s for illusory forms to emerge for various flicker frequencies (Becker & Elliott, 2006). It is, therefore, possible that the 3-s intervals of dynamic noise in Pilot Exp. A were not sufficiently long to induce illusions, whereas the continuous dynamic noise of Exp. 2a maximally enhanced illusory experiences.

Given that dynamic noise can have these different effects on perception, there are two alternative explanations as to why the dynamic white noise in Exp. 2b was not conducive to illusory experience: the first possibility is that the flicker effect of the white noise was not noticeable, and therefore, greatly reduced flicker-enhanced illusory experience. The second alternative is that the objectively lower contrast of the white noise compared to the Gaussian noise induced a luminance interference effect. To expand on this latter point, it is possible that the luminance of the display may have interfered with imagery, and therefore, the relationship between imagery and pareidolia was reduced. A previous study found that image generation (and to a lesser extent, retention) can be disrupted by a sudden change in display luminance (Sherwood & Pearson, 2010). However, as with the DVN, the white noise in our study appeared for a continuous duration of 5.5 min, making this explanation unlikely. Although it is possible that a constant luminant environment generally reduces imagery vividness compared to a dark environment (Thaler et al., 2014), evidence for this is scarce and inconsistent (Narchal & Broota, 1988). Many studies of visual imagery have used highly luminant backgrounds, particularly those that relied on projectors in earlier decades. Kosslyn et al. (1984), for one, used a projector screen as a reference frame for various imagery tasks (e.g., subjects were required to imagine a picture within the bounds of the screen, or to indicate the size or location of an imagined picture by holding their fingers up to the screen). They found that performance on many of these tasks correlated with self-reported imagery vividness, suggesting intact individual variation in imagery abilities despite constant luminance. Therefore, visual interference is less likely a general effect of viewing a luminant background, but rather viewing a change in background luminance at a critical time window. Nevertheless, effects of background luminance will need to be investigated more thoroughly in future studies.

### Pareidolia proneness and perceptual uncertainty

In Exp. 2, we added confidence ratings to responses and analyzed the relationship between pareidolia proneness and perceptual uncertainty. In Exp. 2a (continuous dynamic Gaussian noise), more pareidolia-prone subjects were less confident that a real face was not simply imagined. Conversely, in Exp. 2b (continuous dynamic white noise), there was no association between pareidolia proneness and confidence in percepts. In other words, it seems that a relatively perceptually ambiguous environment is necessary for uncertainty in percepts to contribute to pareidolia experiences, similar to the effects found for imagery vividness. To speculate on the reason for this effect, we consider the interaction between perceptual confidence and perceptual ambiguity. In Exp. 2a, the high-contrast noise led to greater perceptual ambiguity, and increased reliance on internally generated images for visual guidance. For people with low confidence in real percepts (who relied less on real stimuli to make a decision), the boundary between imagery and reality narrowed, leading to more misperceptions. In Exp. 2b, the low-contrast noise led to less perceptual ambiguity, lessening the reliance on internally generated images for visual processing. This broadened the boundary between imagery and reality, and abolished the effect of perceptual confidence on misperception responses.

### Pareidolia as prediction error

Our findings on the contribution of confidence to pareidolia proneness support the interpretation of pareidolia as a reality discrimination issue, particularly as a form of prediction error. In the predictive coding framework, perception is described as a constant prediction-updating process, by which top-down and bottom-up signals interact to reduce uncertainty and prediction error, and form an individual’s optimal version of reality (de Lange et al., 2018; Gordon et al., 2017; Król & El-Deredy, 2011). Increasing noise (uncertainty) in the environment increases prediction error, because the individual must rely more heavily on top-down processes for perception, and this can induce illusory experience (Fermüller & Malm, 2004). Based on our results on imagery vividness, people with more vivid imagery may already rely heavily on top-down processes for perception, which makes them naturally more prone to prediction error. Therefore, the combination of perceptual uncertainty and vivid imagery is associated with enhanced prediction error, which boosts anomalous perceptual experiences.

### Individual differences in perceptual experience

The current study demonstrates a range of individual differences in anomalous perceptual experiences across a normative sample. It is important to investigate these differences, and factors that may contribute to them, for two main reasons: (1) to understand the boundary between normal and abnormal (clinical) perceptual differences, and (2) to determine the efficacy of normative studies in predicting more severe symptoms of perceptual differences, in the absence of pathology.

On the first point, we must do away with the idea that “normal” perception is a consistent, reliable, homogeneous process, and instead move toward the concept of a “normal spectrum” that is differently influenced by top-down mental representations and bottom-up sensory input (Reeder, 2017). An increased awareness of perceptual differences will ultimately allow people to embrace their unique sensory experiences, and may even improve quality of life.

We must emphasize that individual differences in mental sensory representations (imagery) and susceptibility to anomalous perception are neither “good” nor “bad”, and our work cannot predict mental illness or any other disorder. What is important to note, is that people on the tail ends of the spectrum of imagery vividness may have much different experiences to one another, and also to people who sit closer to the middle of the spectrum. Vivid imagery may be detrimental to one’s quality of life only if there is a comorbid pathology: for example, it may coincide with enhanced intrusive imagery in post-traumatic stress disorder (Pearson & Westbrook, 2015) or hallucinations in schizophrenia (Aleman et al., 2000). On the other hand, synaesthesia (a non-clinical condition that involves illusory sensations and is associated with vivid imagery) is reported to be a neutral, or even enjoyable, experience (Rich et al., 2005). At the other tail end, having weak or no imagery may contribute to fewer anomalous experiences, but it is also sometimes associated with heightened (though non-pathological) issues with: working and long-term memory, face recognition, navigation, and even language comprehension (Bergen et al., 2007; Jacobs et al., 2018; Keogh & Pearson, 2018; Watkins, 2018). It is as yet unknown how low (or no) imagery may affect anomalous perception in pathology. In the current study, we did not seek to recruit the “tail-ends” of imagery vividness, and as a result, we did not have any individuals who reported a lack of imagery in our sample (see Supplementary Material: *13. VVIQ analysis*). This would be important to explore in future studies.

On the second point: a few previous studies have found a link between hallucination prevalence and pareidolia. Hallucinating patients with dementia with Lewy bodies (DLB) see significantly more faces in noise than non-hallucinating patients (Mamiya et al., 2016), and patients with DLB report more pareidolia experiences than patients with Alzheimer’s Disease or healthy controls (Uchiyama et al., 2012). The authors hypothesized that these results may reflect a combination of attention deficits and visual dysfunctions in DLB that contribute to susceptibility to both hallucinations and pareidolia. In line with this, both pareidolia and hallucinatory experiences can be reduced in patients with DLB following cholinergic enhancement (Yokoi et al., 2014). These findings provide support for the Perception and Attention Deficit (PAD) model of hallucinations (Collerton et al., 2005). Nevertheless, it is unclear from these studies whether currently hallucination-prone patients were prone to pareidolia already prior to their pathology, or whether pareidolia proneness developed along with hallucinations in these various disorders—a critical distinction. The pharmacological manipulations of previous studies point toward a pathological origin of pareidolia in hallucinating patients (Uchiyama et al., 2012; Yokoi et al., 2014), in favor of the PAD model. However, studies of normative samples (including the current study), favor an interpretation based on prediction error (Smailes et al., 2020). In line with this, clinical studies have demonstrated that some kinds of hallucinations may be explained as a misattribution of imagery as reality (Aleman et al., 2000; Aynsworth et al., 2017; Shine et al., 2015). It is, therefore, important to investigate pareidolia proneness in the absence of (or prior to) pathology, to better understand the relationship between normal and abnormal anomalous perception.

## Electronic supplementary material

Below is the link to the electronic supplementary material.

Supplementary file1 (DOCX 1042 kb)

Supplementary file2 (DOCX 8 kb)

## Footnotes

1 Our experiments and references all focused on the visual domain, so we can only make conclusions based on visual imagery and visual pareidolia. We are aware that imagery, hallucinations, and pareidolia also occur in the auditory domain (e.g., Moseley et al., 2018; Nees & Phillips, 2015), so it is likely that our results would generalize to other, perhaps multiple, senses. This would have to be investigated in future studies.
