# Supplementary material for: Anomalous visual experience is linked to perceptual uncertainty and visual imagery vividness
Source: Psychol Res. 2020 May 31;85(5):1848–65. doi: 10.1007/s00426-020-01364-7 (PMC8289756; doi:10.1007/s00426-020-01364-7)

***1. Environment and testing time analysis***

The MaXLab contained 30 computer stations facing a window and 9 soundproof booths facing away from a window. Blinds were closed during the experiment and soundproof booth doors remained open, with internal lights turned off. This was done in an attempt to control the brightness of the room for all subjects, but there was some natural variability. For each experiment conducted in the MaXLab (Exp.1a, Exp.1b, Pilot Exp.A) we conducted Bayesian (Cauchy prior = 0.707) Mann-Whitney U tests augmented with 5 chains of 1000 iterations in JASP (JASP Team, 2019) to determine if there was any observable effect of the different testing environments (soundproof booth or computer station) on pareidolia proneness. Analyses revealed no evidence for any difference in pareidolia proneness between testing environments (Exp.1a: U = 124.000, BF_10_ = 0.357; Exp.1b: U = 164.000, BF_10_ = 0.382; Pilot Exp.A: U = 108.500, BF_10_ = 0.807). We also conducted Bayesian ANOVAs for each experiment to determine whether there was any effect of the time of day (late morning to late afternoon) and day on which the experiment was performed (three testing days). There was no effect of testing day (Exp.1a: P(M|data) = 0.310, BF_10_ = 0.450; Exp.1b: P(M|data) = 0.218, BF_10_ = 0.279; Pilot Exp.A: P(M|data) = 0.232, BF_10_ = 0.301) or time of day (Exp.1a: P(M|data) = 0.278, BF_10_ = 0.385; Exp.1b: P(M|data) = 0.300, BF_10_ = 0.429; Pilot Exp.A: P(M|data) = 0.260, BF_10_ = 0.351) on pareidolia proneness.

***2. Noise parameters***

Table S1. Blob sizes (measured as the standard deviation (SD) of the 2D Gaussian function) in pixels and degrees of visual angle, with corresponding relative quantities across the noise image.

| SD (in pixels) | SD (app. visual angle) | Relative quantity (%) |
| --- | --- | --- |
| 32 | 0° 50’ (Exp.1-2)  0° 42’ (Exp.3) | 10 |
| 16 | 0° 25’ (Exp.1-2)  0° 21’ (Exp.3) | 30 |
| 10 | 0° 15’ (Exp.1-2)  0° 13’ (Exp.3) | 60 |

Figure S1. a.) On the left: an example of a Gaussian noise image used in Experiments 1-3a (x - and y- axis scale in degrees of visual angel). On the right: corresponding power spectral density vs. spatial frequency in cycles per degree of visual angle (radial average of the 2D Fourier-transform of the image). b.) On the left: an example of a white noise image used in Experiment 3b (x - and y- axis scale in degrees of visual angel). On the right: corresponding power spectral density vs. spatial frequency in cycles per degree of visual angle (radial average of the 2D Fourier-transform of the image).


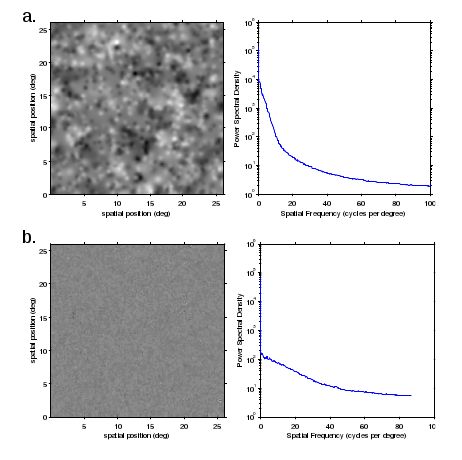


***3. Experiment instructions***

In all experiments, students first read and signed a consent form. In Experiments 1-2, subjects were then given verbal instructions to fill in all demographics information carefully. Here they could choose between a German or English language preference. They were told to choose the language they are most comfortable with because instructions and questionnaires would be in that language. Subjects were told to read experiment instructions carefully, to try their best, and that the experimenter would know if they were not paying attention to the task. The experimenter then took any questions and subjects were instructed to press any key to continue to the demographics form. This collected subject ID, date of birth, gender, handedness, field of study, and language preference. Clicking “OK” took them to a written task instruction screen.

Note that “1” and “2” keys were actually the “b” and “n” keys (centered above the space bar on the keyboard), relabeled with stickers.

*Exp.1a*

Training instructions: *In this experiment you will look for faces in briefly presented noise images. Faces will always appear in the center of the screen so keep your attention focused on the center. If there is a face, press "1". If there is no face, press "2", as soon as the image disappears. There are 6 practice blocks. The first two blocks will be the easiest, to demonstrate what the faces look like. The faces will become increasingly more difficult to perceive over time. Faces will always appear on 50% of trials, randomly mixed within a block. Press any key to proceed.*

Main experiment instructions: *In the main experiment there are 4 longer blocks of trials. All faces will be at or near perceptual threshold, but they will still appear 50% of the time. Press "1" if there was a face and "2" if there was no face, as soon as the image disappears. Sometimes you will see a checkerboard pattern instead of faces in noise- simply ignore these and wait for the next trial. Press any key to proceed.*

*Exp.1b*

Training instructions (additions to Exp.1a in bold): *In this experiment you will look for faces in briefly presented noise images. Faces will always appear in the center of the screen so keep your attention focused on the center. If there is a face, press "1". If there is no face, press "2", as soon as the image disappears. There are 6 practice blocks. The first two blocks will be the easiest, to demonstrate what the faces look like. The faces will become increasingly more difficult to perceive over time.* ***If you do not see a face for a while, or never see a face for a whole block or more, that is not a problem. Only press "1" if you think you may have actually seen a face.*** *Press any key to proceed.*

Main experiment instructions (additions to Exp.1a in bold): *In the main experiment there are 4 longer blocks of trials. If there is a face, press "1". If there is no face, press "2", as soon as the image disappears.* ***If you do not see a face for a while, or never see a face for a whole block or more, that is not a problem. Only press "1" if you think you may have actually seen a face.*** *Sometimes you will see a checkerboard pattern instead of faces in noise- simply ignore these and wait for the next trial. Press any key to proceed.*

*Pilot Exp.A*

Training instructions (changes from Exp.1b in bold): ***In this experiment you will look for faces in moving noise patterns.*** *Faces will always appear in the center of the screen so keep your attention focused on the center.* ***The noise patterns will appear for 3 seconds each. A face can appear at any time during the 3 seconds.*** *If there is a face, press "1". If there is no face, press "2", as soon as the pattern disappears. There are 6 practice blocks. The first two blocks will be the easiest, to demonstrate what the faces look like. The faces will become increasingly more difficult to perceive over time. If you do not see a face for a while, or never see a face, that is no problem. Only press "1" if you think you may have actually seen a face. Press any key to proceed.*

Main experiment instructions were the same as in Exp.1b.

*Experiment 3a and 3b*

Verbal Instructions: *Every so often a face will appear in the noise. The face can appear at or near visual threshold, so it will be very difficult to detect. I do not expect you to see every face. The face can appear at varying times, in various locations, and different sizes and contrasts. It may be more easy to detect sometimes and impossible at other times. Please respond liberally: even if you think you may have seen the impression of a face but are not sure, press the “1” key. Once the confidence screen appears, you can then report how confident you are in your response.*

*The confidence screen appears for only 2 seconds, so you are not expected to read the responses every time. What you need to know is that “1” means you made a mistake in pressing the detection key, “4” means you saw a distinct face, and “2” and “3” mean probably didn't/probably did see a face, but you are unsure. Just remember that responses more toward the left are less confident, and more toward the right are more confident.*

*Respond as much or as little as necessary, just whenever you think you may have seen a face. You can use both hands or one hand to respond, whatever is most comfortable for you. Please respond as soon as you think you have seen a face. Do not deliberate with your response.*

***4. Questionnaires***

VIVIDNESS OF VISUAL IMAGERY QUESTIONNAIRE (VVIQ)

For each item on this questionnaire, try to form a visual image, and consider your experience carefully. For any image that you do experience, rate how vivid it is using the five-point scale described below. If you do not have a visual image, rate vividness as ‘1’. Only use ‘5’ for images that are truly as lively and vivid as real seeing. Please note that there are no right or wrong answers to the questions, and that it is not necessarily desirable to experience imagery or, if you do, to have more vivid imagery.

Perfectly clear and vivid as real seeing 5

Clear and reasonably vivid 4

Moderately clear and lively 3

Vague and dim 2

No image at all, you only “know” that you are

thinking of the object 1

For items 1-4, think of some relative or friend whom you frequently see (but who is not with you at present) and consider carefully the picture that comes before your mind’s eye.

1. The exact contour of face, head, shoulders and body _______________

2. Characteristic poses of head, attitudes of body etc. _______________

3. The precise gait, length of step etc., in walking _______________

4. The different colours of some familiar clothes _______________

Visualise a rising sun. Consider carefully the picture that comes before your mind’s eye.

5. The sun rising above the horizon into a hazy sky _______________

6. The sky clears and surrounds the sun with blueness _______________

7. Clouds. A storm blows up with flashes of lightning _______________

8. A rainbow appears _______________

Think of the front of a shop which you often go to. Consider the picture that comes before your mind’s eye.

9. The overall appearance of the shop from the opposite side

of the road _______________

10. A window display including colours, shapes and details

of individual items for sale _______________

11. You are near the entrance. The colour, shape and

details of the door. _______________

12. You enter the shop and go to the counter. The counter

assistant serves you. Money changes hands _______________

Finally think of a country scene which involves trees, mountains and a lake. Consider the picture that comes before your mind’s eye.

13. The contours of the landscape _______________

14. The colour and shape of the trees _______________

15. The colour and shape of the lake _______________

16. A strong wind blows on the trees and on the lake causing

waves in the water. _______________

**Faces Questionnaire**

**Please read each question and circle the answer(s) that best describes what you experienced. If your answer is not available, please write it down in the available space.**

**1. About how many faces did you see in one run?**

0-5 5-10 15-20 50-100 Other

**2. About how long did each face appear?**

Brief flash ~1 second ~3 seconds Other

**3. How much of the face was usually visible?**

The whole face One half of the face (e.g., top/bottom/left/right) Other

Some parts (e.g., eyes/eyebrows/mouth/nose) Vague shape

**4. Where did the face tend to appear on screen?**

Near the center More on the left/right/top/bottom Far from the center

All over the screen Center Other

**5. About how big was the face?**

Variable The size of a fingerprint The size of a post-it note It filled much of the screen

Other

**6. About how frequently did you see the faces?**

Sometimes one right after the other within a couple of seconds Every 1-2 seconds

Every 5-10 seconds Every 1-2 minutes Variable Other

***5. Group-average results***

*Exp.1a*

We conducted group-average analyses to compare our results to those of Zhang et al. (2008). Zhang and colleagues reported face-present responses on 35.52% of trials with a standard deviation (SD) of 14% (*N* = 16). Subjects in our experiment (*N* = 42) were generally more conservative with their face-present responses, with a mean of 13.26% (SD = 17.47%). The reason for this discrepancy was largely due to subjects withholding responses on many trials (mean = 27.87%, SD = 28.24%), rather than reporting to have seen no face – a result that was not anticipated (but see above in *Exploring non-responses*).

Subjects made face-absent responses on 58.86% (SD = 28.75%) of trials on average. This result suggests that generally subjects knew that they should have seen no face about 50% of the time, although the SD suggests a high amount of variability in subject responses. was A Bayesian paired-samples t-test conducted in JASP revealed extremely strong evidence for a discrepancy between the number of face-absent responses and face-present responses (BF_10_ = 7.122e+6), which suggests that subjects were generally more likely to respond if they did not see a face compared to when they did see a face. This result seems to suggest that if subjects were unsure about the presence of a face (as reported in *Exploring non-responses*), they simply did not respond. Non-responses from indecisive subjects, coupled with non-responses that reflected a true lack of illusory experience, led to a much lower number of face-present responses overall.

*Exp.1b*

In Exp.1b, subjects responded that they saw faces on 5.69% (SD = 7.92%) of trials on average. A Bayesian Mann-Whitney U test (data augmentation algorithm with 5 chains and 1000 iterations, Cauchy prior = 0.707) revealed anecdotal evidence that this proportion was different from that reported in Exp.1a (U = 581.500, BF_10_ = 1.731), suggesting a small influence of instructions on responses. Again, subjects chose to withhold responses on many trials (mean = 32.79%, SD = 31.39%), and we found no evidence for a difference in the proportion of non-responses between experiments (U = 812.500, BF_10_ = 0.241). There was furthermore no evidence for a difference in the proportion of face-absent responses in Experiment 1b (mean = 61.53%, SD = 31.01%) compared to Exp.1a (U = 841.500, BF_10_ = 0.301), and the SD again indicates a high amount of individual variability in responses. As in Exp.1a, a Bayesian paired-samples t-test showed an extremely strong difference between the number of face-absent and face-present responses (BF_10_ = 1.534e+10).

The addition of an instruction than puts less pressure on subjects to respond positively slightly reduced positive responses overall. However, this did not decrease non-responses, which may indicate continued hesitancy to respond when subjects were unsure about the presence of a face.

*Pilot Exp.A*

In Pilot Exp.A, subjects made a face-present response on 5.53% (SD = 10.04%) of trials on average. A Bayesian Mann-Whitney U test revealed anecdotal evidence that this was different from the face-present responses reported in Exp.1a (U = 1013.000, BF_10_ = 2.023), and no evidence for a difference from the face-present responses reported in Exp.1b (U = 741.000, BF_10_ = 0.275). We found extremely strong evidence that subjects chose to withhold responses on a lower proportion of trials (mean = 4.95%, SD = 12.19%) than in the previous experiment (Exp.1a: U = 1368.500, BF_10_ = 2300.819; Exp.1b: U = 1156.500, BF_10_ = 234.033). We also found extremely strong evidence that subjects made a higher proportion of face-absent responses (mean = 88.52%, SD = 15.05%) compared to Exp.1a (U = 215.000, BF_10_ = 1030.166) and Exp.1b (U = 264.500, BF_10_ = 136.805). The change from static to short dynamic noise led to an extreme reduction of non-responses compared to Exp.1, which indicates less response uncertainty in Pilot Exp.A. Subjects also made more face-absent responses, suggesting short dynamic noise is not conducive to pareidolia illusions.

*Exp.2a*

On average, subjects reported to have seen 66.63 real faces (SD = 15.83) corresponding to 47.59% of the real faces presented in the main experiment (SD = 13.19%). A Bayesian one-sample t-test performed on the group mean hit rate (47.59%) compared to the target hit rate of 50%, revealed no evidence for a difference (BF_10_ = 0.327, error % = 0.036). This indicates that face detection was near perceptual threshold for most participants. Subjects reported fewer misperceptions than detections (mean = 23.26, SD = 28.37). A Bayesian paired-samples t-test revealed extremely strong evidence for a true difference (BF_10_ = 773.638). Face detections were furthermore reported with higher confidence (mean = 3.47, SD = 0.33) than misperceptions (mean = 2.53, SD = 0.40; BF_10_ = 3.269e+9). Note that this latter analysis was performed only for subjects who reported both detections and misperceptions (N=25).

*Exp.2b*

Subjects reported to have seen 45.57 real faces (SD = 29.36), corresponding to 32.55% of the real faces presented in the main experiment (SD = 20.97%). A Bayesian one-sample t-test performed on the group mean hit rate (32.55%) compared to the target hit rate of 50%, revealed very strong evidence for a difference (BF_10_ = 54.251, error % = 9.181e-5). This indicates that faces appeared below perceptual threshold for most participants. A Bayesian Mann-Whitney U test revealed anecdotal evidence for a difference in the number of real faces detected between Exp.2a and Exp.2b (U = 162.000, BF_10_ = 2.696). Subjects reported fewer misperceptions than detections (mean = 19.96, SD = 23.04), which was no different from the number of misperceptions reported in Exp.2a (U = 409.000, BF_10_ = 0.285). This suggests that although real faces were slightly more difficult to perceive in this experiment, it did not affect the average rate of misperceptions. A Bayesian paired-samples t-test revealed strong evidence for a difference between the number of face detections and misperceptions (BF_10_ = 29.604), similar to Exp.2a.

Face detections were furthermore reported with higher confidence (mean = 2.96, SD = 0.52) than misperceptions (mean = 2.45, SD = 0.39; BF_10_ = 261.500). Note that this analysis was performed only for subjects who reported both detections and misperceptions. Bayesian Mann-Whitney U tests showed strong evidence that confidence in real face detections was significantly lower than in Exp.2a (U = 193.000, BF_10_ = 13.777), reflecting greater perceptual uncertainty, but confidence in misperceptions was roughly the same (U = 255.000, BF_10_ = 0.356). This indicates that lower confidence in face detections did not affect confidence in misperceptions.

***6. Exploring non-responses***

An unanticipated finding was the lack of responses on many trials in Exp.1a (mean = 27.87%, SD = 28.24%). When prompted by e-mail as to why most subjects did not respond on some trials, many reported that they were hesitant to respond if they were unsure about the presence of a face in the noise images, or that they did not have enough time to decide (they were given 1200 ms), and that the task was difficult. Although we cannot directly interpret non-responses, these reports suggest that the majority of subjects remained naive to the true nature of the experiment: that no faces actually appeared. Only one participant from Exp.1a (excluded from analysis for another reason) wrote explicitly that he was aware no faces appeared in the experiment. Furthermore, these anecdotes suggest that non-responses may have reflected a lack of confidence in percepts, which could explain why subjects thought they needed more time to make a decision and found the task difficult (it was, after all, a simple detection task).

A two-tailed Bayesian Kendall’s τ**b** correlation analysis revealed extremely strong evidence for a negative relationship between the number of non-responses and face-absent responses for both Exp.1a (τ**b** = -0.600, BF_10_ = 781727.501, 95% CIs = [-0.749, -0.359]) and Exp.1b (τ**b** = -0.852, BF_10_ = 5.802e+10, 95% CIs = [-0.928, -0.561]). Subjects who made more face-absent responses (that is, those who were non-pareidolia prone) had fewer non-responses. This suggests that they were more confident that no face was present, since they did not deliberate about their responses.

Nevertheless, there is a more trivial explanation for the non-responses: it is possible that because no actual faces appeared in the main experiment, there was a decrease in response motivation. Those who saw more faces (and people with more vivid imagery), may have simply been more motivated to respond generally. To test this, we performed correlations between number of face-present responses and total responses, and between imagery vividness and total responses. For the former analysis, we found anecdotal evidence for a positive relationship in both in Exp.1a (τ**b** = 0.198, BF_10_ = 1.057, 95% CIs = [-0.014, 0.383]) and Exp.1b (τ**b** = 0.233, BF_10_ = 1.567, 95% CIs = [0.004, 0.425]), suggesting a negligible relationship between faces seen and total responses. We furthermore found moderate evidence for a negative relationship between imagery vividness and total number of responses in both Exp.1a (τ**b** = -0.297, BF_10_ = 8.388, 95% CIs = [-0.477, -0.080]) and Exp.1b (τ**b** = -0.297, BF_10_ = 5.446, 95% CIs = [-0.487, -0.064]), further enforcing our hypothesis that people with more vivid imagery were more uncertain in their percepts, rather than more motivated to respond. However, because we cannot directly interpret response profiles (we do not know if non-responses reflect uncertainty or boredom), the relationship between pareidolia proneness, perceptual uncertainty, and motivation were explored directly in Exp.2.

To ensure that all subjects had an adequate number of responses for analyses, we sought to exclude subjects whose total number of responses fell more than 2.5 standard deviations below the mean number of total responses. Subjects who fell under this criterion had already been excluded based on criteria stated in the main manuscript, and no further subjects were excluded from Exp.1a or Exp.1b.

***7. Proportions vs. Probability***

We analyzed binomial probability instead of raw proportions because probabilities not only took into account the percentage of faces seen, but also the likelihood of achieving this value compared to the expected value (0.39). This added variance to the data because seeing faces on, for example, 1/50 responses was much more likely than seeing faces on 1/400 responses (therefore penalizing the high face-absent/low face-present responders as being less pareidolia prone). Here is a visual example of the 10 subjects who made fewer than five face-present responses:

Individual subjects are plotted along the x-axis, and the graph on the left shows the proportion of face-present to face-absent responses, and the graph on the right shows log-transformed probability values. The two graphs follow a similar trajectory except where subjects made an especially low or high number of face-absent responses – here I will use the example of subject 3 (0/49 present-to-absent responses) and subjects 5-7 (1/350+ present-to-absent responses). The proportion of present-to-absent responses is of course lower for the subject who made 0 face-present responses (subject 3, with 0 coded as 0.01 so proportions and probabilities could be calculated) compared to the subjects who made just 1 face-present response for 350+ face-absent responses (subjects 5-7). However, it is more probable that subject 3 (who made 0 face-present responses and 49 face-absent responses) is pareidolia prone compared to the subjects who made 1 face-present response for 350+ face-absent responses (subjects 5-7). Therefore, the proportion of face-present responses is higher for subjects 5-7, but the probability of being pareidolia prone is lower. This measure therefore better reflects pareidolia proneness, because we hypothesized that subjects who had more non-responses were more pareidolia prone and were less likely to report faces due to uncertainty rather than not actually seeing faces.

***8. Qualitative results***

For all experiments, immediately following the main task, subjects filled out a questionnaire about the qualities of the faces they saw in the noise. Our goal was to obtain richer details about these purely subjective experiences. Subjects were asked to report the approximate number, frequency, duration, size, location, and clarity of features of faces they saw in the main experiment, and they were given the option to write in any other information they wished to provide that was not covered by the available choices.

Responses were coded with a number that indicated the scale of subjective perceptions (reported in Table S2 and S3): number (low-to-high), frequency (low-to-high), size (small-to-big), duration (short-to-long), location (center-to-periphery), clarity of features (vague-to-whole face). Bayesian Kendall’s tau-b (τ**b**) correlations were performed between these responses. Analyses were only performed for Exp.1 and Pilot Exp.A, since subjective experiences in Exp.2 could be based on both actual face perceptions and misperceptions, and these cannot be disentangled. Analyses were performed on the combined qualitative data of Exp.1a and Exp.1b because the stimuli were the same in both experiments.

*8.1 Exp.1*

The full summary of responses is reported in Table S2 (also see Figure S2 for an illustrative summary of qualitative reports). Perceiving faces in varied locations stood out as a strong predictor of perceiving other illusory features that deviated from features that appeared on training trials. We found extremely strong evidence for a positive correlation between the perceived location of faces and perceived number (τ**b** = 0.346, BF_10_ = 3278.155), size (τ**b** = 0.396, BF_10_ = 75584.178), and clarity of features of faces (τ**b** = 0.354, BF_10_ = 5205.062); that is, seeing faces in less central and more varied locations was strongly associated with perceiving a higher number of larger faces with more complete features. There was also a strong correlation between perceived location and duration of faces (τ**b** = 0.238, BF_10_ = 17.018), and a moderate correlation between perceived location and frequency of faces (τ**b** = 0.211, BF_10_ = 6.040). Furthermore, we found extremely strong evidence for a positive correlation between perceived size and the number of faces seen (τ**b** = 0.335, BF_10_ = 1813.982) and strong evidence for a positive association between perceived size and duration of faces (τ**b** = 0.252, BF_10_ = 30.168). Finally, we found moderate evidence for a positive correlation between perceived frequency of faces and the clarity of facial features (τ**b** = 0.199, BF_10_ = 4.117).

--Table S2 about here--

Figure S2. An illustrative summary of subjective reports. Subjects who reported to see fewer faces tended to see them smaller , with fewer features, and more centrally. Subjects who reported to see more faces tended to see them larger, more clearly, and in variable locations around the screen. Below each depictive representation is a selected list of self-reported phrases subjects used to describe their experiences.


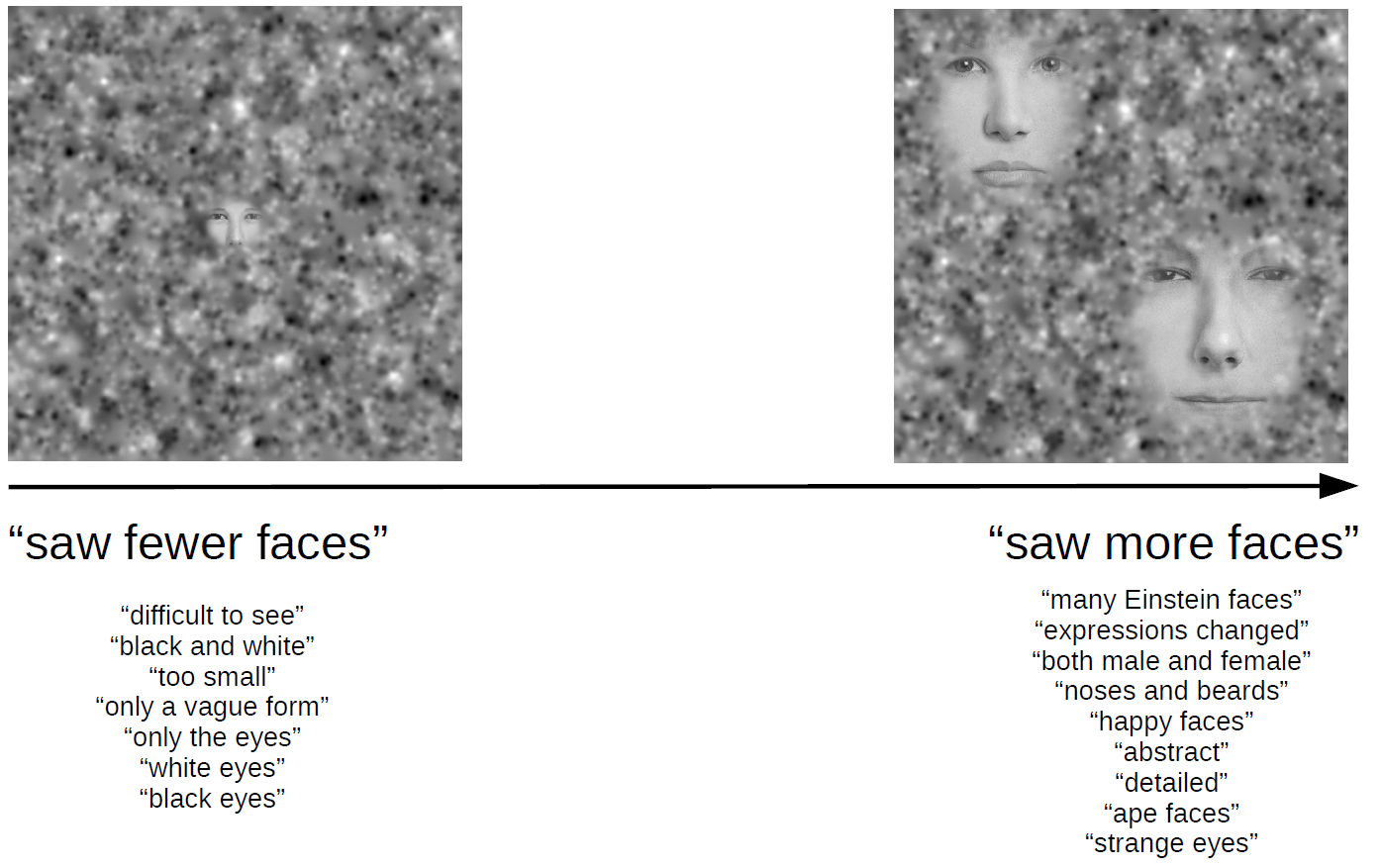


*8.2 Pilot Exp.A*

The full summary of responses is reported in Table S3. Despite generally more conservative responses in this experiment compared to Exp.1, and no evidence for a correlation between pareidolia proneness and imagery vividness, we continued to find several correlations between qualitative responses. Particularly, perceived location of faces again stood out as a predictor of many other illusory features. We found extremely strong evidence for a positive correlation between perceived location and size of faces (τ**b** = 0.419, BF_10_ = 135.268). We also found very strong evidence for a positive correlation between perceived location and duration (τ**b** = 0.382, BF_10_ = 45.116), as well as strong evidence for a correlation between both perceived location and number (τ**b** = 0.348, BF_10_ = 18.216) and location and frequency of faces (τ**b** = 0.345, BF_10_ = 16.891). This suggests that subjects who saw faces in less central and more varied locations tended to see more (and more frequent) larger faces that persisted for a longer amount of time. We also found strong evidence for a positive correlation between perceived size and duration (τ**b** = 0.359, BF_10_ = 24.421), and moderate evidence for a correlation between size and number (τ**b** = 0.297, BF_10_ = 5.429). Finally, we found moderate evidence for a correlation between perceived clarity of features and face duration (τ**b** = 0.289, BF_10_ = 4.613).

--Table S3 about here--

***9. Bottom-up analyses***

*9.1 Additional classification analyses*

Figure S3. The results of the reverse correlation analysis as described in *3.2 Behavioral classification analyses*, showing the mean median correlation of ROIs for Exp.1a (a) and Exp.1b (b). Errors bars represent ± 1 standard error from the mean.


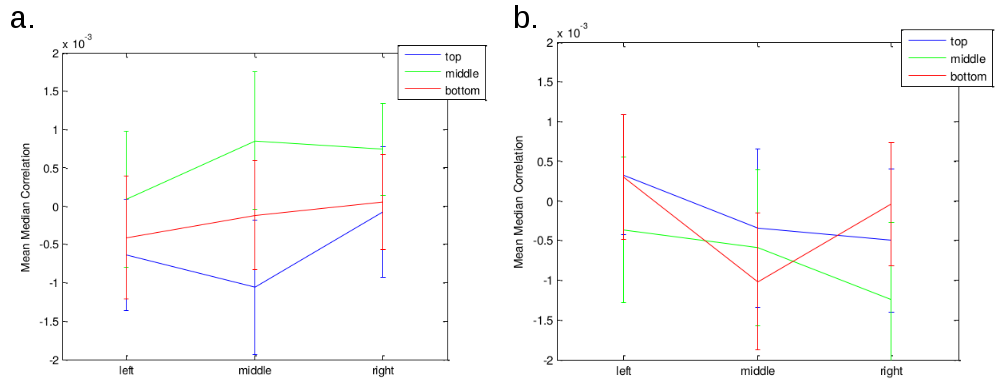


Table S4. A summary of the t-tests performed on the correlations within the different ROIs in the within-subjects CIs as reported in *3.2 Behavioral classification analyses*. a.) Exp.1a. b.) Exp.1b.

| **a.** | Estimate | Std. Error | df | t-value | Pr(<\|t\|) |
| --- | --- | --- | --- | --- | --- |
| (Intercept) | -6.38E-04 | 7.81E-04 | 369 | -0.817 | 0.625 |
| Top Middle | -4.21E-04 | 1.10E-03 | 369 | -0.381 | 0.703 |
| Top Right | 5.64E-04 | 1.10E-03 | 369 | 0.511 | 0.609 |
| Middle Left | 7.27E-04 | 1.10E-03 | 369 | 0.659 | 0.511 |
| Center | 1.49E-03 | 1.10E-03 | 369 | 1.35 | 0.178 |
| Middle Right | 1.38E-03 | 1.10E-03 | 369 | 1.249 | 0.212 |
| Bottom Left | 2.24E-04 | 1.10E-03 | 369 | 0.203 | 0.84 |
| Bottom Middle | 5.18E-04 | 1.10E-03 | 369 | 0.203 | 0.639 |
| Bottom Right | 6.90E-04 | 1.10E-03 | 369 | 0.625 | 0.532 |

| **b.** | Estimate | Std. Error | df | t-value | Pr(>\|t\|) |
| --- | --- | --- | --- | --- | --- |
| (Intercept) | 3.27E-04 | 8.86E-04 | 323.1 | 0.369 | 0.713 |
| Top Middle | -6.72E-04 | 1.24E-03 | 288 | -0.541 | 0.589 |
| Top Right | -8.27E-04 | 1.24E-03 | 288 | -0.666 | 0.589 |
| Middle Left | -6.91E-04 | 1.24E-03 | 288 | -0.556 | 0.578 |
| Center | -9.16E-04 | 1.24E-03 | 288 | -0.737 | 0.461 |
| Middle Right | -1.57E-03 | 1.24E-03 | 288 | -1.262 | 0.208 |
| Bottom Left | -2.48E-05 | 1.24E-03 | 288 | -0.02 | 0.984 |
| Bottom Middle | -1.35E-03 | 1.24E-03 | 288 | -1.086 | 0.279 |
| Bottom Right | -3.70E-04 | 1.24E-03 | 288 | -0.298 | 0.766 |

Table S5. A summary of the percentage of z-scores in the top 1% of scores contained in the between-subjects CI. Note that these are unthresholded scores that do not pass multiple comparison correction.

|  | Exp.1a | | Exp.1b | |
| --- | --- | --- | --- | --- |
|  | z > 2.39 | z < -2.20 | z > 2.31 | z < -2.39 |
| Top Left | 0.9 | 0.9 | 0.8 | 0.5 |
| Top Middle | 1.7 | 1.3 | 0.5 | 1.0 |
| Top Right | 0.4 | 1.3 | 2.2 | 0.2 |
| Middle Left | 0.9 | 1.4 | 0.9 | 1.1 |
| Center | 0.9 | 0.8 | 1.7 | 1.3 |
| Middle Right | 0.5 | 1.3 | 0.6 | 0.6 |
| Bottom Left | 1.4 | 1.0 | 0.4 | 2.1 |
| Bottom Middle | 1.2 | 0.4 | 0.7 | 1.8 |
| Bottom Right | 1.2 | 0.7 | 1.2 | 0. |
| Whole Frame | 1 | 1 | 1 | 1 |

In addition to the behavioral classification analyses on the Gaussian noise images, we compared the mean luminance of Gaussian noise images that elicited face-present vs. face-absent responses in Exp.1a and Exp.1b separately. Mean luminance and the Root Mean Square Contrast (CRMS) were calculated for each of the 480 noise images. Analyses were performed for the entire image, as well as for nine ROIs of equal size (defined by overlaying a 3 x 3 grid over each noise image), to determine if any low-level differences were tied to specific locations. To determine whether the noise parameters had different effects on subjects depending on the vividness of their visual imagery, subjects in each experiment were further median split into two groups according to VVIQ score (low-VVIQ, high-VVIQ; Exp.1a: median = 4.03; Exp.1b: median = 3.88). The significance threshold for the full-image and ROI analyses was Bonferroni-corrected to *p* < 0.05/10 tests = 0.005.

We performed 10 t-tests (comparing images that elicited face-present and face-absent responses) for mean differences in CRMS and luminance in Exp.1a and Exp.1b for subjects with high and low VVIQ. Regardless of the test performed, we found no significant differences between images that elicited face-present and face-absent responses (see Figure S4, and Table S6 for a summary of the t-tests performed).

Figure S4. Aggregated noise luminance for subjects with high vs. low VVIQ on images that elicited face-absent and face-present responses in Exp.1a (a) and Exp.1b (b).


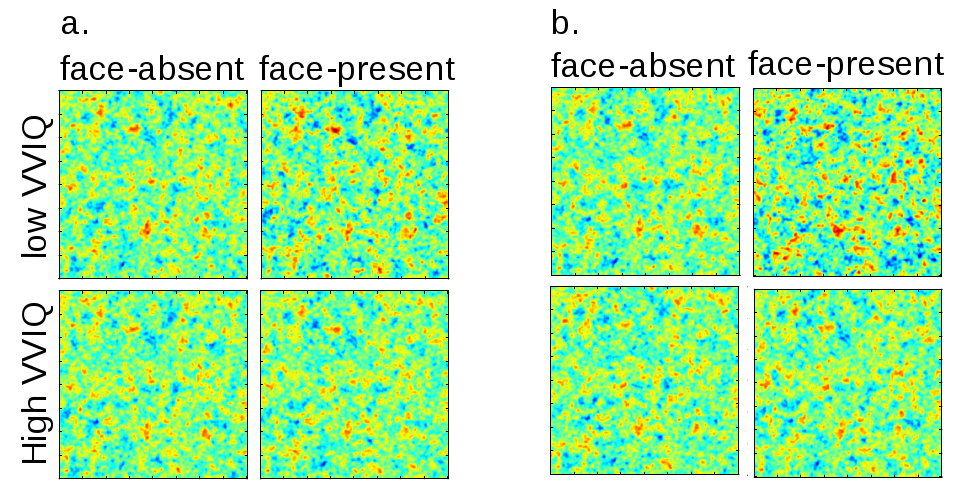


Table S6. All of the t-tests performed for Exp.1a and Exp.1b on mean differences between images that elicited face-present and face-absent responses. The number of face-absent images was larger than the number of face-present images. To equalize the number of images in our analyses, we chose randomly from the list of face-absent images so that the same number of images were input for face-absent and face-present responses (the total number of images in each analysis, as well as the original number of face-absent images, are shown below each sub-table).

**Exp.1a Luminance VVIQ < 4.03**

| Region | df | t-value | *p* |
| --- | --- | --- | --- |
| Top Left | 6782 | 0.233 | 0.81 |
| Top Middle | 6782 | 1.360 | 0.17 |
| Top Right | 6782 | -0.391 | 0.70 |
| Middle Left | 6782 | 1.248 | 0.21 |
| Center | 6782 | -0.274 | 0.78 |
| Middle Right | 6782 | 0.411 | 0.68 |
| Bottom Left | 6782 | -1.768 | 0.08 |
| Bottom Middle | 6782 | 0.995 | 0.32 |
| Bottom Right | 6782 | 0.304 | 0.76 |
| Whole Frame | 6782 | 0.152 | 0.87 |

3392 samples from former 6688 face-absent images

**Exp.1a Luminance VVIQ > 4.03**

| Region | df | t-value | *p* |
| --- | --- | --- | --- |
| Top Left | 9800 | 0.902 | 0.38 |
| Top Middle | 9800 | 0.212 | 0.83 |
| Top Right | 9800 | 0.125 | 0.90 |
| Middle Left | 9800 | 0.028 | 0.98 |
| Center | 9800 | -0.865 | 0.39 |
| Middle Right | 9800 | -0.901 | 0.37 |
| Bottom Left | 9800 | 0.254 | 0.80 |
| Bottom Middle | 9800 | 0.357 | 0.72 |
| Bottom Right | 9800 | 0.502 | 0.62 |
| Whole Frame | 9800 | -1.249 | 0.21 |

4901 samples from former 5179 face-absent images

**Exp.1a CRMS VVIQ < 4.03**

| Region | df | t-value | *p* |
| --- | --- | --- | --- |
| Top Left | 6782 | 2.104 | 0.04 |
| Top Middle | 6782 | 1.710 | 0.09 |
| Top Right | 6782 | -0.173 | 0.86 |
| Middle Left | 6782 | -0.990 | 0.32 |
| Center | 6782 | 0.859 | 0.39 |
| Middle Right | 6782 | -1.129 | 0.25 |
| Bottom Left | 6782 | 1.210 | 0.23 |
| Bottom Middle | 6782 | -0.948 | 0.34 |
| Bottom Right | 6782 | 0.303 | 0.76 |
| Whole Frame | 6782 | 1.648 | 0.10 |

3392 samples from former 6688 face-absent images

**Exp.1a CRMS VVIQ > 4.03**

| Region | df | t-value | *p* |
| --- | --- | --- | --- |
| Top Left | 9800 | -0.966 | 0.33 |
| Top Middle | 9800 | 0.721 | 0.47 |
| Top Right | 9800 | -0.411 | 0.68 |
| Middle Left | 9800 | -0.248 | 0.80 |
| Center | 9800 | -0.641 | 0.52 |
| Middle Right | 9800 | 0.0618 | 0.95 |
| Bottom Left | 9800 | -1.241 | 0.21 |
| Bottom Middle | 9800 | 0.961 | 0.34 |
| Bottom Right | 9800 | 0.109 | 0.91 |
| Whole Frame | 9800 | 0.368 | 0.71 |

4901 samples from former 5179 face-absent images

**Exp.1b Luminance VVIQ < 3.88**

| Region | df | t-value | *p* |
| --- | --- | --- | --- |
| Top Left | 5580 | -0.080 | 0.94 |
| Top Middle | 5580 | 0.909 | 0.36 |
| Top Right | 5580 | 1.490 | 0.13 |
| Middle Left | 5580 | -0.108 | 0.91 |
| Center | 5580 | 0.007 | 0.99 |
| Middle Right | 5580 | -1.040 | 0.30 |
| Bottom Left | 5580 | 1.847 | 0.06 |
| Bottom Middle | 5580 | 0.700 | 0.48 |
| Bottom Right | 5580 | -0.965 | 0.33 |
| Whole Frame | 5580 | 0.331 | 0.74 |

2791 samples from former 6329 face-absent images

**Exp.1b Luminance VVIQ >3.88**

| Region | df | t-value | *p* |
| --- | --- | --- | --- |
| Top Left | 8082 | 0.074 | 0.94 |
| Top Middle | 8082 | 0.056 | 0.96 |
| Top Right | 8082 | 0.109 | 0.91 |
| Middle Left | 8082 | -0.689 | 0.49 |
| Center | 8082 | 0.957 | 0.34 |
| Middle Right | 8082 | 0.193 | 0.85 |
| Bottom Left | 8082 | -0.452 | 0.65 |
| Bottom Middle | 8082 | 1.062 | 0.29 |
| Bottom Right | 8082 | -1.109 | 0.27 |
| Whole Frame | 8082 | -0.583 | 0.56 |

4042 samples from former 4598 face-absent images

**Exp.1b CRMS VVIQ < 3.88**

| Region | df | t-value | *p* |
| --- | --- | --- | --- |
| Top Left | 5466 | 1.675 | 0.09 |
| Top Middle | 5466 | 1.537 | 0.12 |
| Top Right | 5466 | 0.096 | 0.92 |
| Middle Left | 5466 | 0.837 | 0.40 |
| Center | 5466 | -0.273 | 0.78 |
| Middle Right | 5466 | -0.055 | 0.95 |
| Bottom Left | 5466 | 0.677 | 0.49 |
| Bottom Middle | 5466 | 0.114 | 0.91 |
| Bottom Right | 5466 | -0.090 | 0.93 |
| Whole Frame | 5466 | 1.169 | 0.24 |

2734 samples from former 5426 face-absent images

**Exp.1b CRMS VVIQ > 3.88**

| Region | df | t-value | *p* |
| --- | --- | --- | --- |
| Top Left | 11116 | -0.082 | 0.93 |
| Top Middle | 11116 | 1.222 | 0.22 |
| Top Right | 11116 | -0.223 | 0.82 |
| Middle Left | 11116 | -0.261 | 0.79 |
| Center | 11116 | -0.225 | 0.82 |
| Middle Right | 11116 | -0.940 | 0.34 |
| Bottom Left | 11116 | -1.254 | 0.20 |
| Bottom Middle | 11116 | 0.507 | 0.61 |
| Bottom Right | 11116 | 0.873 | 0.38 |
| Whole Frame | 11116 | 0.738 | 0.46 |

5559 samples from former 6441 face-absent images

*9.2 A tally of pareidolia experiences*

Another method of determining bottom-up influences on responses is to perform a simple tally of face-present responses for each noise image. Each subject in Exp.1a and Exp.1b saw each of the 480 Gaussian noise images once. We counted the number of face-present responses made for each image across Exp.1 and analyzed the distribution of responses. This revealed a range between 22-46 face-present responses (mean = 34.72, median = 35, SD = 3.88) per image. There were two images (each with 46 face-present responses) that fell outside 2.5 SD from the mean number of responses. We hypothesized that these two images do not adequately contribute to the mean number of faces seen or their standard deviations (Exp.1a: mean = 63.65, SD = 83.80, Exp.1b: mean = 27.31, SD = 38.01, see Supplementary Material: *5. Group-average results*). To test this, we performed an analysis in which we removed two face-present responses from all subjects who reported two or more face-present responses (under the hypothetical assumption that all subjects reported seeing faces in the two images in question). We then re-ran our correlation analyses between pareidolia proneness and imagery vividness for Exp.1a and Exp.1b. This revealed no categorical change in our results, although numerically the correlation became slightly stronger for Exp.1a and slightly weaker for Exp.1b, the latter of which was likely due to the smaller number of face-present responses overall for Exp.1b (Exp1.a: τ**b** = 0.323, BF+0 = 33.070, 95% CIs = [0.104, 0.499]; Exp.1b: τ**b** = 0.172, BF+0 = 1.182, 95% CIs = [0.016, 0.375]).

***10. Pilot experiment A: Short dynamic noise***

To test whether dynamic noise boosts pareidolia experiences, in this pilot we presented static images that changed randomly every ~67 ms within a 3-second time window. 45 images appeared in quick succession in a single trial, which created the illusion of a “dynamic” display of Gaussian noise.

***10.1 Methods***

*10.1.1 Subjects*

49 subjects were recruited via the MaXLab online recruitment tool. All data were collected over three testing days, and were analyzed following complete data collection. 12 subjects were excluded due to failing to pass the attention check (N=9) or having incomplete data (N=3). We had a final group size of 37. Subjects were reimbursed for 10 euros for the 1-hour experiment, as per laboratory guidelines.

We obtained a diverse sample of bachelor's and master's students studying engineering, business/economics, social science, mathematics, medicine, sport science, computer science, media/journalism, psychology, education, and natural sciences. Our sample included 8 women, 0 left-handed, 7 English speakers, and 30 German speakers, with a mean age of 24.00 years (range = 20-30). All subjects provided written, informed consent to take part in the experiment. The experiment was approved by the Otto-von-Guericke University ethics committee and adhered to the tenets of the Declaration of Helsinki.

*10.1.2 Apparatus*

Pilot Exp.A took place at the same times and in the same environments as Exp.1. There was no general effect of environment on subject performance (see Supplementary Material: *1. Environment analysis*).

*10.1.3 Stimuli*

Stimuli were the same as those used in Exp.1. Static Gaussian noise images were chosen randomly from the same set of 480 images, and appeared in a string of 45 images presented for ~67 ms each, for a total presentation time of 3 seconds on every trial.

*10.1.4 Procedure*

The experimental procedure was similar to Exp.1. Like Exp.1b, instructions did not pressure subjects to respond on a certain proportion of trials. Furthermore, the instructions now told subjects to look for faces in moving noise patterns that would appear for 3 seconds at a time (see Supplementary Material: *3. Experiment instructions*). Faces in the training trials appeared for a duration of 600 ms, 1800 ms after the onset of the dynamic noise. This setup encouraged two expectations for face detections in the main experiment: 1.) that faces would appear for 600 ms rather than ~67 ms (the duration of a single static noise image), discouraging subjects from focusing on a single static noise image for face detection; and 2.) that faces would appear somewhere temporally in the middle of the dynamic noise presentation. This encouraged subjects to attend to the dynamic noise for the full 3 seconds.

The instructions presented just prior to the main experiment were the same as in Exp.1b. No faces appeared in the main experiment, following the procedure of the previous experiments. For this experiment, we were interested to investigate the effects of removing static, face-like, bottom-up information on pareidolia proneness in subjects with different imagery vividness.

***10.2 Results***

The group-average results pointed toward a highly homogeneous dataset compared to the previous experiment (with a much higher proportion of face-absent responses (mean = 88.52%, SD = 15.05%) compared to Exp.1a (*t*(77) = 5.82, *p* < 0.001) and 1b (*t*(72) = 5.05, *p* < 0.001); see Supplementary Material: *5. Group-average results*).

Pareidolia proneness and imagery vividness were calculated in the same way as in Exp.1. One-tailed Kendall’s τ**b** correlations and BF analyses conducted in JASP revealed virtually no relationship between pareidolia proneness and imagery vividness (τ**b**= -0.118, BF_+0_ = 0.110). The results rather provide moderate evidence for a true null relationship (BF_0+_ = 9.066).

Additional qualitative analyses performed on the data from Pilot Exp.A can be found in the Supplementary Material: *7. Qualitative results*, and Table S3.

***10.3 Discussion***

Pilot Exp.A was conducted to determine whether presenting Gaussian noise as a dynamic display increases perceptual ambiguity and enhances the experience of pareidolia. However, this design instead abolished the relationship between pareidolia proneness and imagery vividness. Does this mean that dynamic noise is generally not conducive to pareidolia experience?

It is possible that three seconds is too brief for illusions to emerge from dynamic noise, even for pareidolia-prone subjects. Early research into the subjective perception of dynamic visual noise suggests that the rapid successive presentation of static noise images leads to the experience of a seemingly continuous display of illusory motion and depth, which can appear to form patterns over time that vary across individuals (Fiorentini & Mackay, 1965; Mackay, 1957). Once we see a display as dynamic rather than static, some kind of temporal pattern may first need to be established before imagined information can emerge. In addition to this, short-duration dynamic noise may actually interfere with imagery (Quinn & McConnell, 1996). Therefore, in Exp.2, we increased our duration of dynamic noise (5.5 minutes) to maximally boost illusory effects.

The results of Pilot Exp.A further led us to speculate on the role of uncertainty in pareidolia experiences: there was no difference between Exp.1b and Pilot Exp.A in the number of face-present responses, but rather in the number of face-absent responses. This led to the hypothesis that subjects who were more pareidolia prone were more uncertain in their responses, and therefore had more non-responses than subjects who were more certain that there were no faces in the display. There is no reason why the number of non-responses should be so much lower in Pilot Exp.A compared to Exp.1, unless these reflect certainty. Subjects had the same amount of time to respond in Pilot Exp.A, so they simply deliberated less about what they had seen. Our hypothesis is therefore that subjects (including pareidolia-prone subjects) were more certain that there were no faces present in Pilot Exp.A, whereas subjects who are pareidolia-prone were less certain of the absence of faces in Exp.1b (hence the higher number of non-responses). To test this, we directly investigated the relationship between perceptual confidence and pareidolia proneness in Exp.2.

***11. Gender analyses***

We performed gender-split analyses on the data of Exp.1a (women = 20; men = 22) and Exp.1b (women = 12; men = 25) separately, to determine if gender differences contributed to our results. We performed Bayesian Mann-Whitney U tests comparing men and women in pareidolia proneness (binomial probabilities) and imagery vividness (mean VVIQ scores) in separate analyses. This revealed no gender differences for either Exp.1a (pareidolia proneness: U = 222.500, BF_10_ = 0.306; imagery vividness: U = 172.500, BF_10_ = 0.483) or Exp.1b (pareidolia proneness: U = 148.000, BF_10_ = 0.345; imagery vividness: U = 162.500, BF_10_ = 0.367).

***12. Perceptual threshold calculation***

*Exp.2a*

At the end of each training run, if subjects saw more than 70% of the real faces, face opacity was lowered by 4% for the following run. If subjects saw between 60-70% of the faces, opacity was lowered by 2%. 40-60% face detections did not change the opacity value. A report of 20-40% faces raised opacity by 2%, and fewer than 20% face detections raised opacity by 4% for the following run. Opacity values were collected on each practice run and the opacity value of the 4^th^ run was used as the starting opacity value in the main experiment. Each run of the main experiment followed the same thresholding procedure as in the practice, to ensure that performance remained close to perceptual threshold.

***13. VVIQ analysis***

Figure S5. Illustrates the distribution of VVIQ scores reported across all experiments (*N* = 174). There was a mean score of 3.767 (SD = 0.637). We conducted a Kolmogorov-Smirnov Test of Normality to determine whether the VVIQ scores reported in our experiments follow a normal distribution. The value of the K-S test statistic (D) was 0.077, *p* = 0.242. This result indicates that our data do not differ significantly from that which is normally distributed.


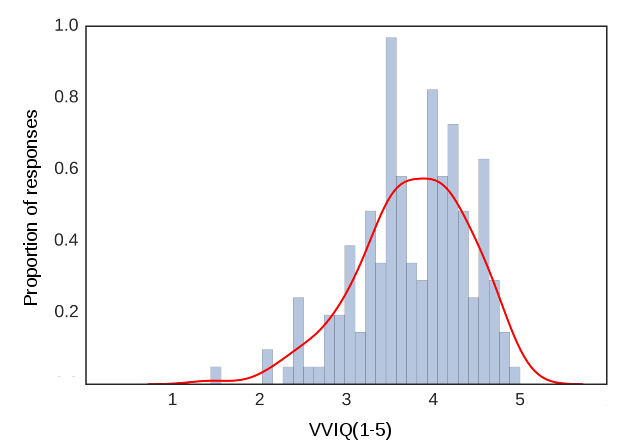

Supplement: Supplementary file 1 — Supplementary file1 (DOCX 1042 kb) [file 426_2020_1364_MOESM1_ESM.docx]
