# Supplementary material for: Anomalous visual experience is linked to perceptual uncertainty and visual imagery vividness
Source: Psychol Res. 2020 May 31;85(5):1848–65. doi: 10.1007/s00426-020-01364-7 (PMC8289756; doi:10.1007/s00426-020-01364-7)
Supplement: Supplementary file 2 — Supplementary file2 (DOCX 8 kb) [file 426_2020_1364_MOESM2_ESM.docx]

Table S2. The results of the face questionnaire from Exp.1 (*N* = 79). Subjects reported the number, frequency, size, location, duration, and clarity of features of the faces they had seen. The sub-heading “Response” indicates the response subjects made. The sub-heading “Code” is the code the experimenter gave the response for Spearman ranking. The sub-heading “Freq” is the frequency (number of subjects) with which that response was made. Subjects could choose a pre-written response, or enter their own response (“Other”). The value of the “Other” response code was manually determined by the experimenter to fit among the other responses. Note that because responses were ranked in the Spearman correlation, the relative difference between values (e.g., 1 vs. 1.5 compared to 2 vs. 3) was not a factor in the analysis.

| Number | | | Frequency | | | Size | | | Location | | | Duration | | | Clarity of features | | |
| --- | --- | --- | --- | --- | --- | --- | --- | --- | --- | --- | --- | --- | --- | --- | --- | --- | --- |
| Response | Code | Freq | Response | Code | Freq | Response | Code | Freq | Response | Code | Freq | Response | Code | Freq | Response | Code | Freq |
| 0-5 | 1 | 49 | None (no faces) | 0 | 15 | None (no faces) | 0 | 9 | None (no faces) | 0 | 7 | None (no faces) | 0 | 3 | None (no faces) | 0 | 11 |
| 5-10 | 2 | 21 | Every 1-2 min. | 1 | 10 | Finger-  print | 1 | 34 | Center | 1 | 49 | Brief flash | 1 | 27 | Vague form | 1 | 15 |
| 15-20 | 3 | 7 | Every 5-10 sec. | 2 | 3 | Post-it | 2 | 11 | Near center | 2 | 0 | ~1 sec. | 2 | 43 | Parts (e.g., eyes) | 2 | 48 |
| 50-100 | 4 | 1 | Every 1-2 sec. | 3 | 7 | Whole screen | 3 | 1 | Bottom/ top/ left/ right | 3 | 1 | ~3 sec. | 3 | 3 | Half face | 3 | 2 |
|  |  |  | More rapid | 4 | 8 |  |  |  |  |  |  |  |  |  | Whole face | 4 | 3 |
| Variable | 6 | 0 | Variable | 6 | 36 | Variable | 6 | 22 | Variable | 6 | 22 | Variable | 6 | 0 | Variable | 6 | 2 |
| Other: | | | Other: | | | Other: | | | Other: | | | Other: | | | Other: | | |
| 5-10 then  50-100 | 3.5 | 1 |  |  |  | Smaller | 0.5 | 2 |  |  |  | ~2 sec. | 2.5 | 1 |  |  |  |

Table S3. The results of the face questionnaire from Pilot Exp.A (*N* = 37). Subjects reported the number, frequency, size, location, duration, and clarity of features of the faces they had seen. The sub-heading “Response” indicates the response subjects made. The sub-heading “Code” is the code the experimenter gave the response for Spearman ranking. The sub-heading “Freq” is the frequency (number of subjects) with which that response was made. Subjects could choose a pre-written response, or enter their own response (“Other”). The value of the “Other” response code was manually determined by the experimenter to fit among the other responses. Note that because responses were ranked in the Spearman correlation, the relative difference between values (e.g., 1 vs. 1.5 compared to 2 vs. 3) was not a factor in the analysis.

| Number | | | Frequency | | | Size | | | Location | | | Duration | | | Clarity of features | | |
| --- | --- | --- | --- | --- | --- | --- | --- | --- | --- | --- | --- | --- | --- | --- | --- | --- | --- |
| Response | Code | Freq | Response | Code | Freq | Response | Code | Freq | Response | Code | Freq | Response | Code | Freq | Response | Code | Freq |
| 0-5 | 1 | 25 | None (no faces) | 0 | 7 | None (no faces) | 0 | 6 | None (no faces) | 0 | 5 | None (no faces) | 0 | 6 | None (no faces) | 0 | 6 |
| 5-10 | 2 | 8 | Every 1-2 min. | 1 | 2 | Finger-  print | 1 | 18 | Center | 1 | 27 | Brief flash | 1 | 13 | Vague form | 1 | 7 |
| 15-20 | 3 | 4 | Every 5-10 sec. | 2 | 2 | Post-it | 2 | 8 | Near center | 2 | 2 | ~1 sec. | 2 | 16 | Parts (e.g., eyes) | 2 | 20 |
| 50-100 | 4 | 0 | Every 1-2 sec. | 3 | 2 | Whole screen | 3 | 0 | Bottom/ top/ left/ right | 3 | 0 | ~3 sec. | 3 | 1 | Half face | 3 | 2 |
|  |  |  | More rapid | 4 | 5 |  |  |  |  |  |  |  |  |  | Whole face | 4 | 1 |
| Variable | 6 | 0 | Variable | 6 | 18 | Variable | 6 | 5 | Variable | 6 | 3 | Variable | 6 | 1 | Variable | 6 | 1 |
| Other: | | | Other: | | | Other: | | | Other: | | | Other: | | | Other: | | |
|  |  |  | Every 2 min. | 0.5 | 1 |  |  |  |  |  |  |  |  |  |  |  |  |
